# Supplementary material for: Structural basis of SIRT7 nucleosome engagement and substrate specificity
Source: Nat Commun. 2025 Feb 4;16:1328. doi: 10.1038/s41467-025-56529-y (PMC11790868; doi:10.1038/s41467-025-56529-y)
Supplement: Supplementary file 1 — Supplementary Information [file 41467_2025_56529_MOESM1_ESM.pdf]

Supplementary Information

Structural basis of SIRT7 nucleosome engagement and substrate specificity

Carlos Moreno-Yruela[1,†,*], Babatunde E. Ekundayo[2,3,†], Polina N. Foteva[1], Dongchun Ni[2,3], Esther Calvino-Sanles[1], Henning Stahlberg[2,3] & Beat Fierz[1,*]

Table of contents

Supplementary Figures 1–13 3

Supplementary Tables 1–5 16

Chemical synthesis 21

General methods 21

Peptide synthesis 21

On-resin lysine modification 23

Peptide cleavage and deprotection 24

Semi-synthesis of modified full-length histone H3 24

Histone expression and purification 26

Expression and purification of H3(29-135) and H3(47-135) constructs 26

Expression and purification of wild type H2A, H2B, H3 and H4 27

Gel electrophoresis of protein, DNA and nucleosome samples 28

Purity traces 30

Mass spectra 32

Supplementary References 35

Supplementary Figures 1–13

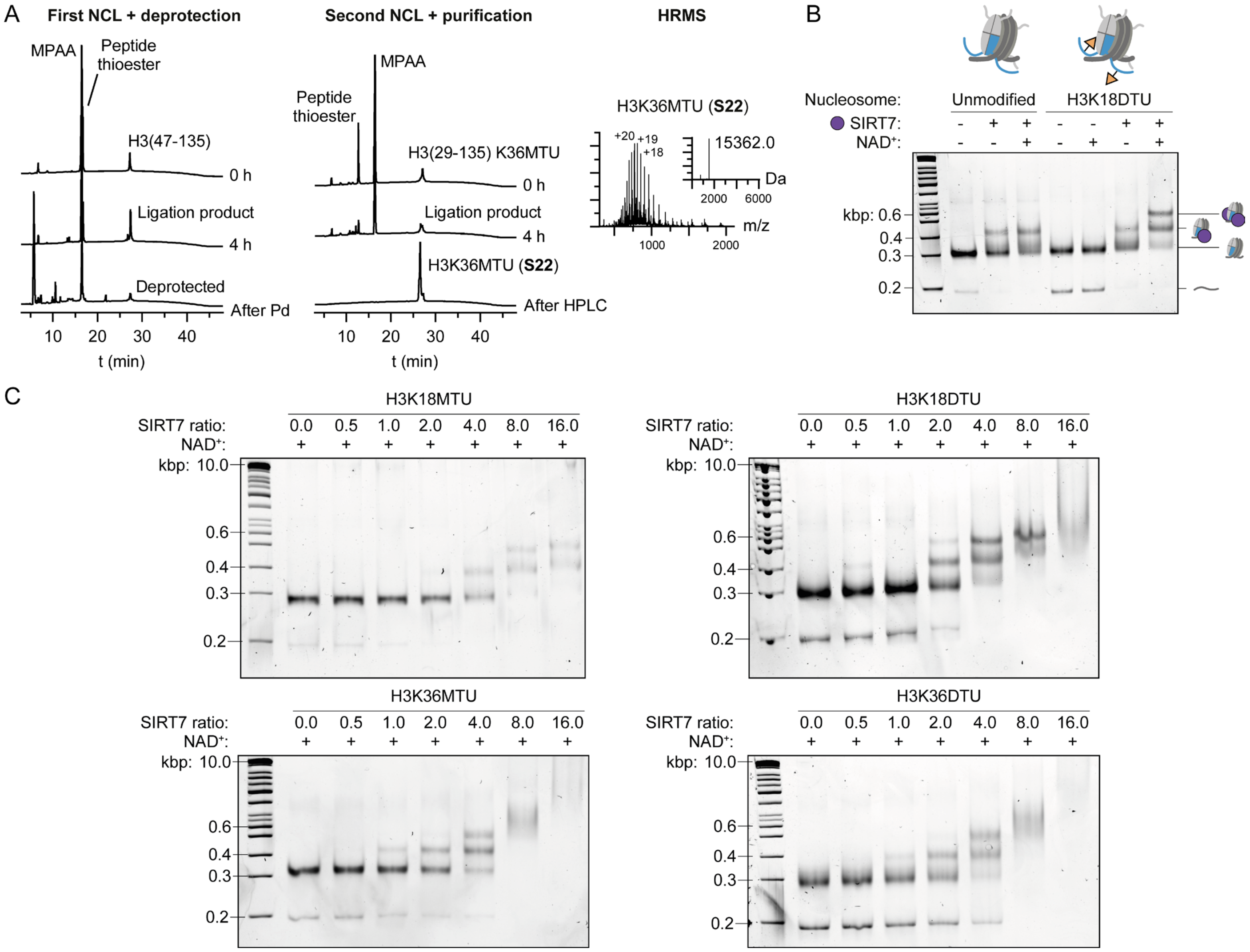

Supplementary Fig. 1. Synthesis and testing of thiourea-functionalized nucleosomes. A, Summary of full length H3K36MTU (**S22**) semi-synthesis. Analytical HPLC traces of the reaction mixture during the first native chemical ligation (NCL) reaction and subsequent thiazolidine deprotection (left), analytical HPLC traces of the reaction mixture during the second NCL reaction and after final purification (center), and final MS spectrum and deconvoluted mass of the final purified product (right). See Supplementary Figs. 18, 19, 22, 23 for complete purity traces and MS spectra of all synthesized histones. **B**, SIRT7 EMSA of nucleosome samples with and without H3K18DTU modification (SIRT7-nucleosome ratio: 3.0). **C**, Representative dose-response EMSAs with each of the four thiourea-containing nucleosomes prepared. Source data are provided as a Source Data file.

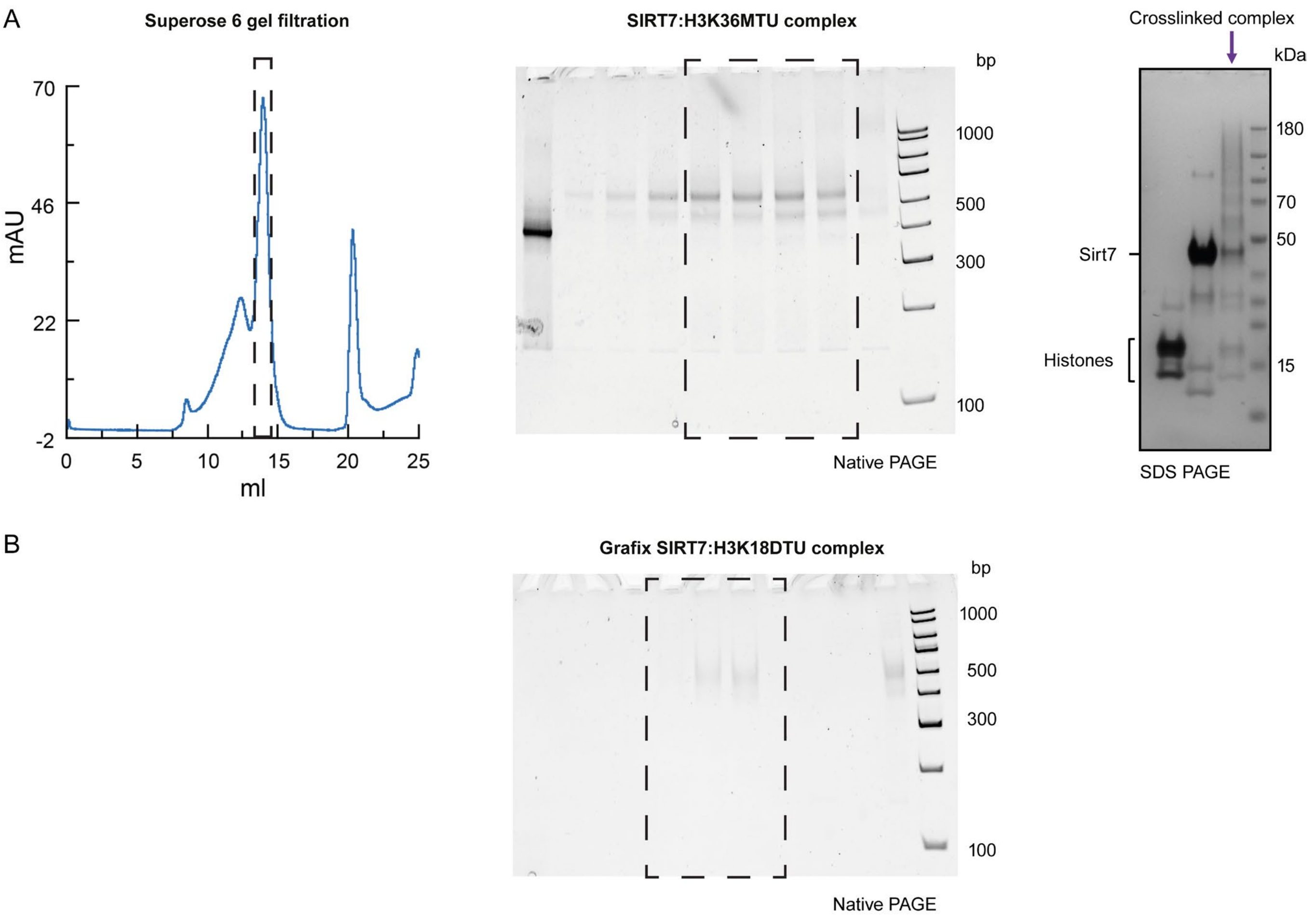

Supplementary Fig. 2. Purification of SIRT7:nucleosome complexes. A, Left panel shows the gel filtration chromatogram from the purification of the crosslinked SIRT7:H3K36-nucleosome complex, the middle panel shows a Native gel of the gel filtration fraction and the right panel shows an SDS-PAGE gel of the pooled crosslinked complex. Dashed lines indicate the fractions pooled for cryo-EM. **B**, Native gel of fractions from Grafix purification of the SIRT7:H3K18DTU nucleosome complex. Dashed lines indicate the fractions pooled for cryo-EM.

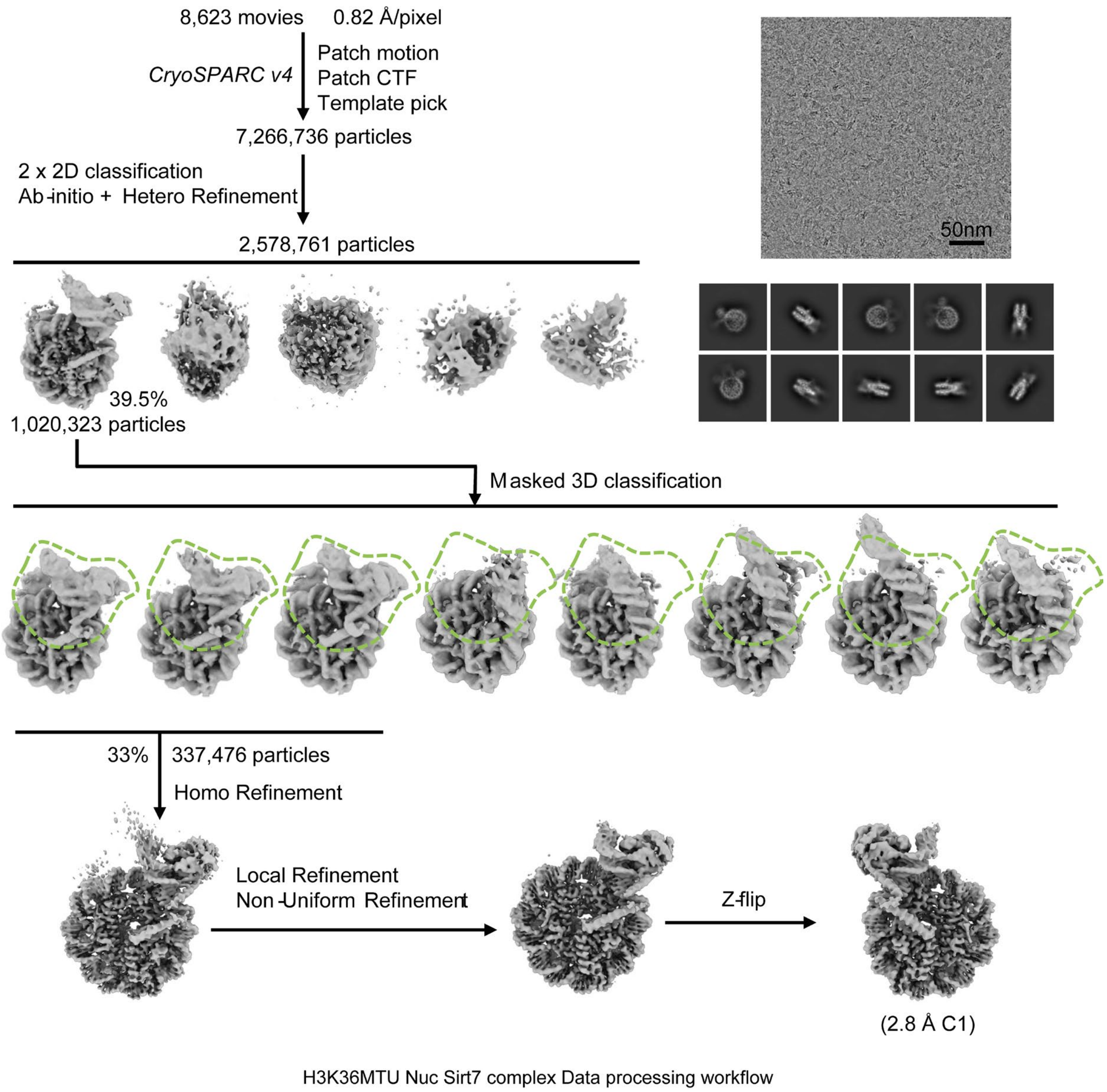

Supplementary Fig. 3. SIRT7:H3K36MTU complex data processing workflow. Processing workflow for SIRT7:H3K36MTU complex showing 2D class averages, 3D classification and refinement steps. Focused classification with the regions covered by the mask are also shown.

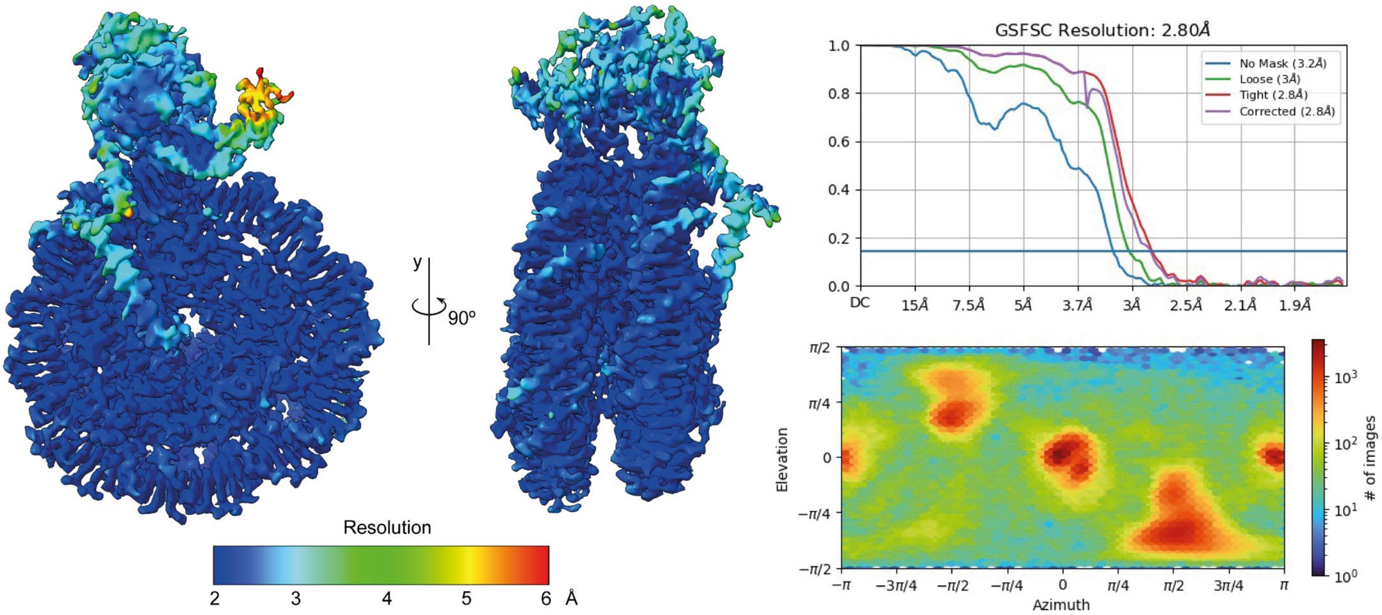

Supplementary Fig. 4. SIRT7:H3K36MTU cryo-EM. Local resolution map, Fourier Shell Correlation (FSC) curve and particle distribution orientation of the SIRT7:H3K36MTU-nucleosome complex.

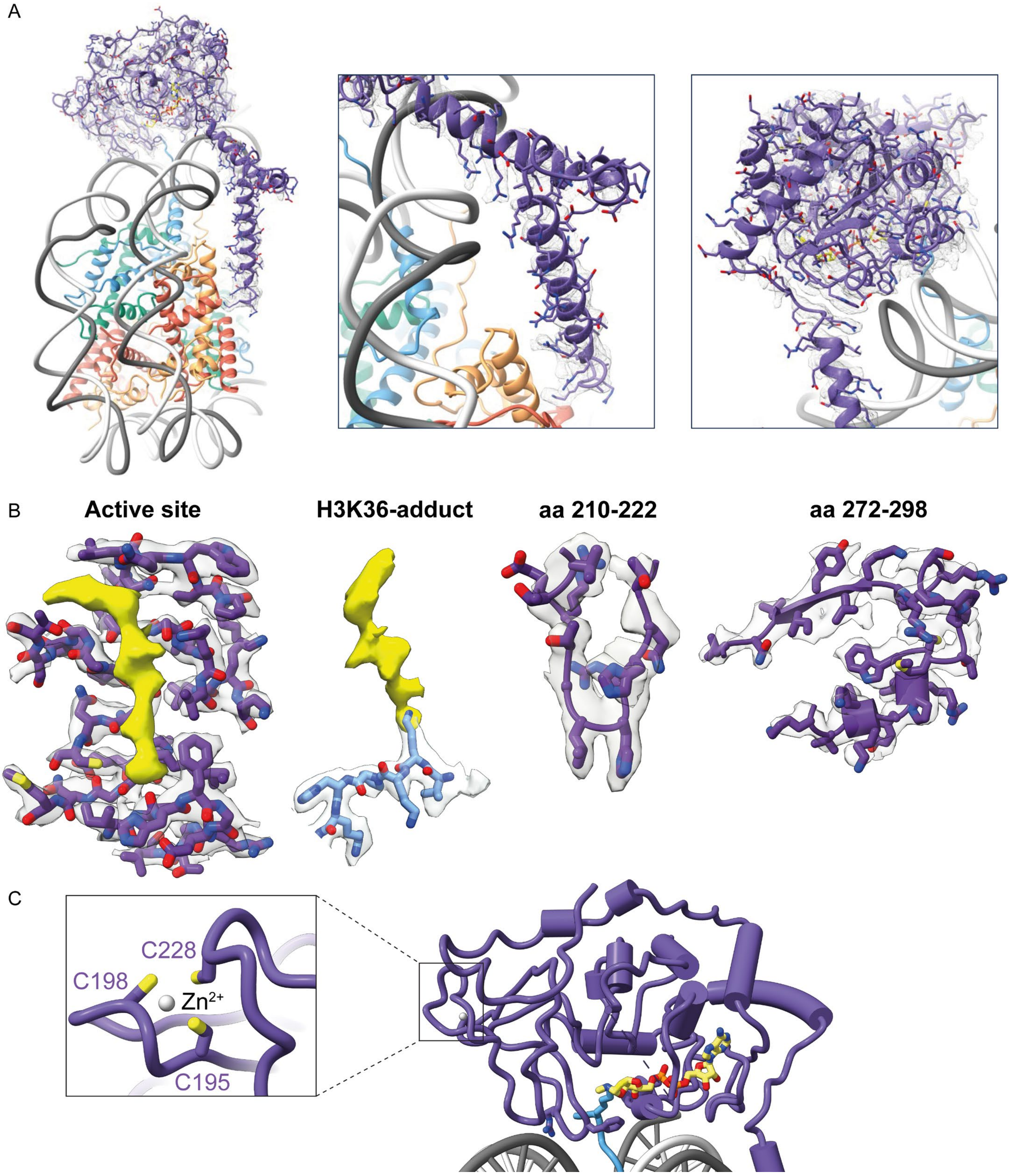

Supplementary Fig. 5. SIRT7:H3K36MTU model quality. A, Overall and zoom-in views of the cryo-EM map of structural elements of SIRT7 in the cryo-EM map (mesh representation) of the SIRT7:H3K36MTU-nucleosome complex. **B**, Detail of side-chain definition in the active site, substrate histone tail, and selected loops. **C**, Detail of the Zn^{2+}-binding residues of the SIRT7 catalytic domain.

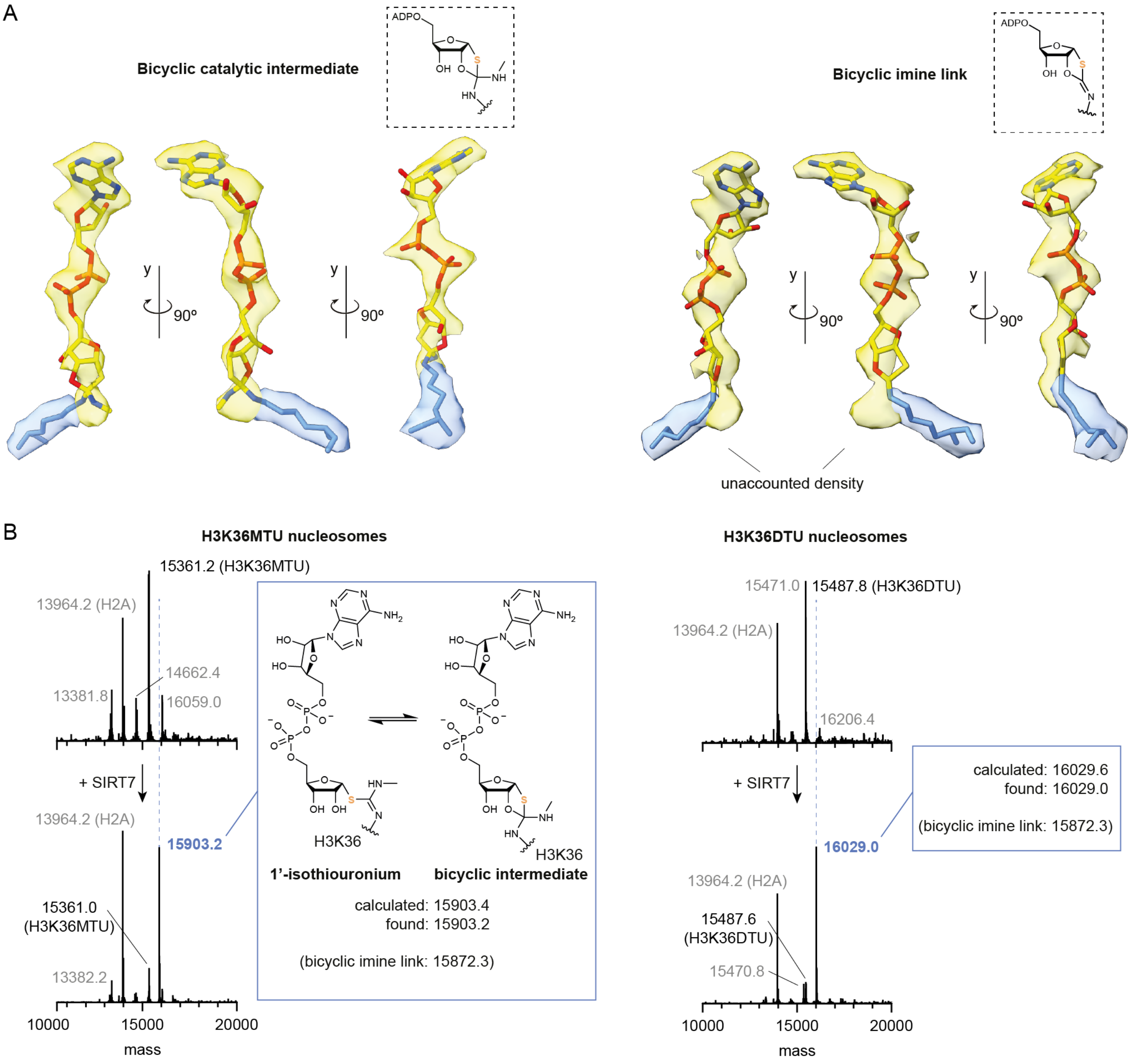

Supplementary Fig. 6. Chemical identity of the mechanism-based crosslink. A, Fitting of a bicyclic catalytic intermediate or a bicyclic imine link model into the H3K36MTU adduct density, and specific density corresponding to the methylamine substituent. Here, our cryo-EM density was less compatible with the imine geometry and clearly indicated that the methylamine substituent is still present. **B**, LC-MS analysis of H3K36MTU and H3K36DTU incubated without (top spectra) and with (bottom spectra) SIRT7, and highlighted mass of the ADPr adducts, which include the methylamine and decylamine substituents, respectively. This analysis shows the methylamine and decylamine substituents likely as 1'-isothiouronium adducts, but does not indicate any imine species. Together, we confirmed that thioureas form the envisioned mechanism-based complexes within the SIRT7 active site via the equilibrium of 1'-isothiouronium and bicyclic catalytic intermediates without amine elimination. Under our LC-MS conditions, H3 co-eluted partially with H2A.

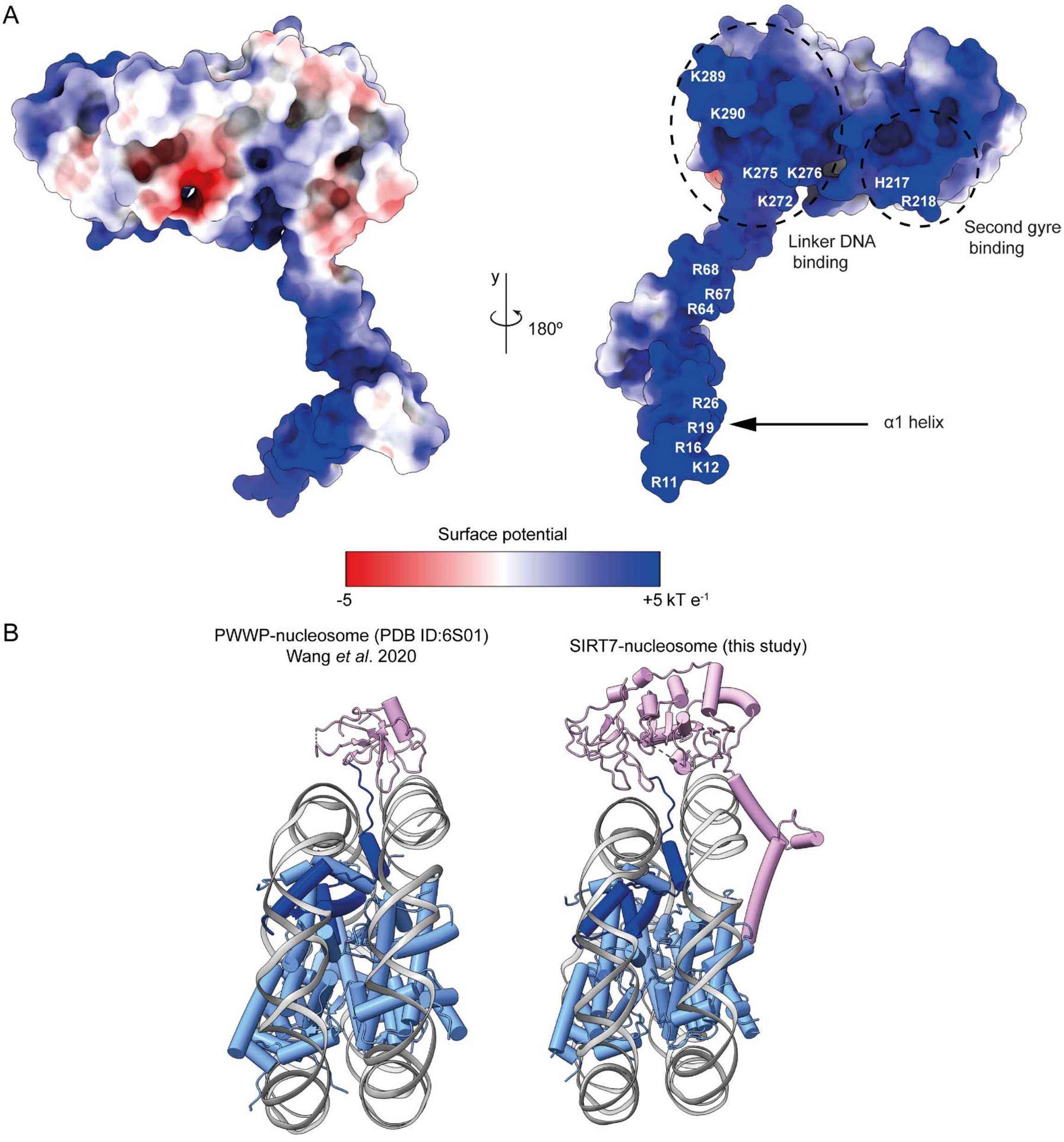

Supplementary Fig. 7. Recognition of H3K36 substrates. A, Surface potential of SIRT7 with highlighted nucleosome-binding regions. **B**, Comparison of nucleosome binding at H3K36 by a PWWP domain (PDB 6S01)[1] and SIRT7.

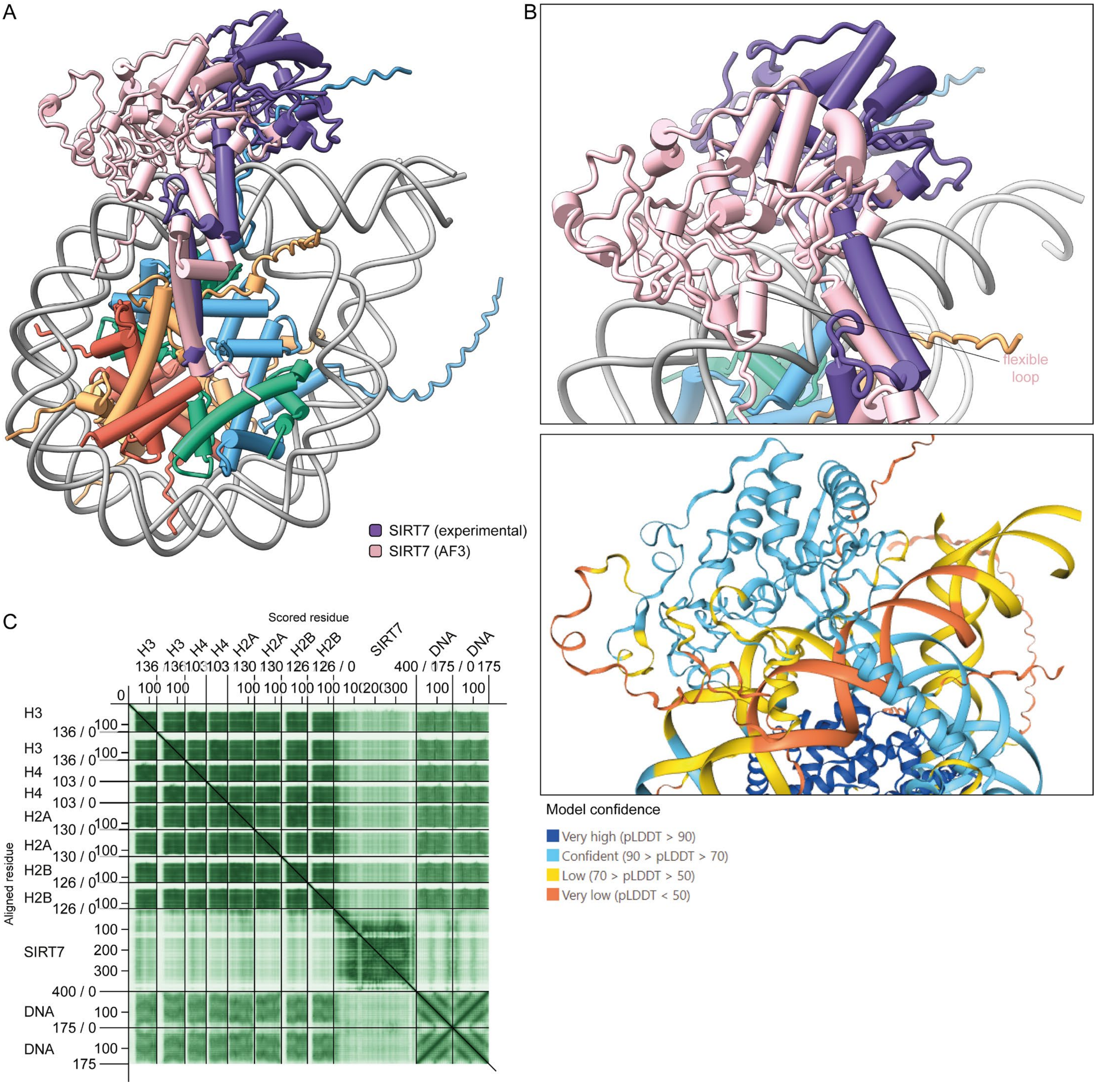

Supplementary Fig. 8. AlphaFold 3 prediction of the SIRT7:nucleosome complex. A, Overlay of the AF3 SIRT7:nucleosome complex and our experimental model of SIRT7 bound to the H3K36MTU nucleosome (not shown). Structures were aligned by the histone octamer. **B**, Detail of the flexible loop within the catalytic domain (aa 120–138) and its AF3-predicted interaction with DNA. This region has lower confidence than the rest of the catalytic domain according to AF3, as seen in the lower panel colored by pLDDT (created with PAE viewer)[2]. **C**, Predicted aligned error (PAE) matrix of the AF3 complex, created with PAE viewer[2].

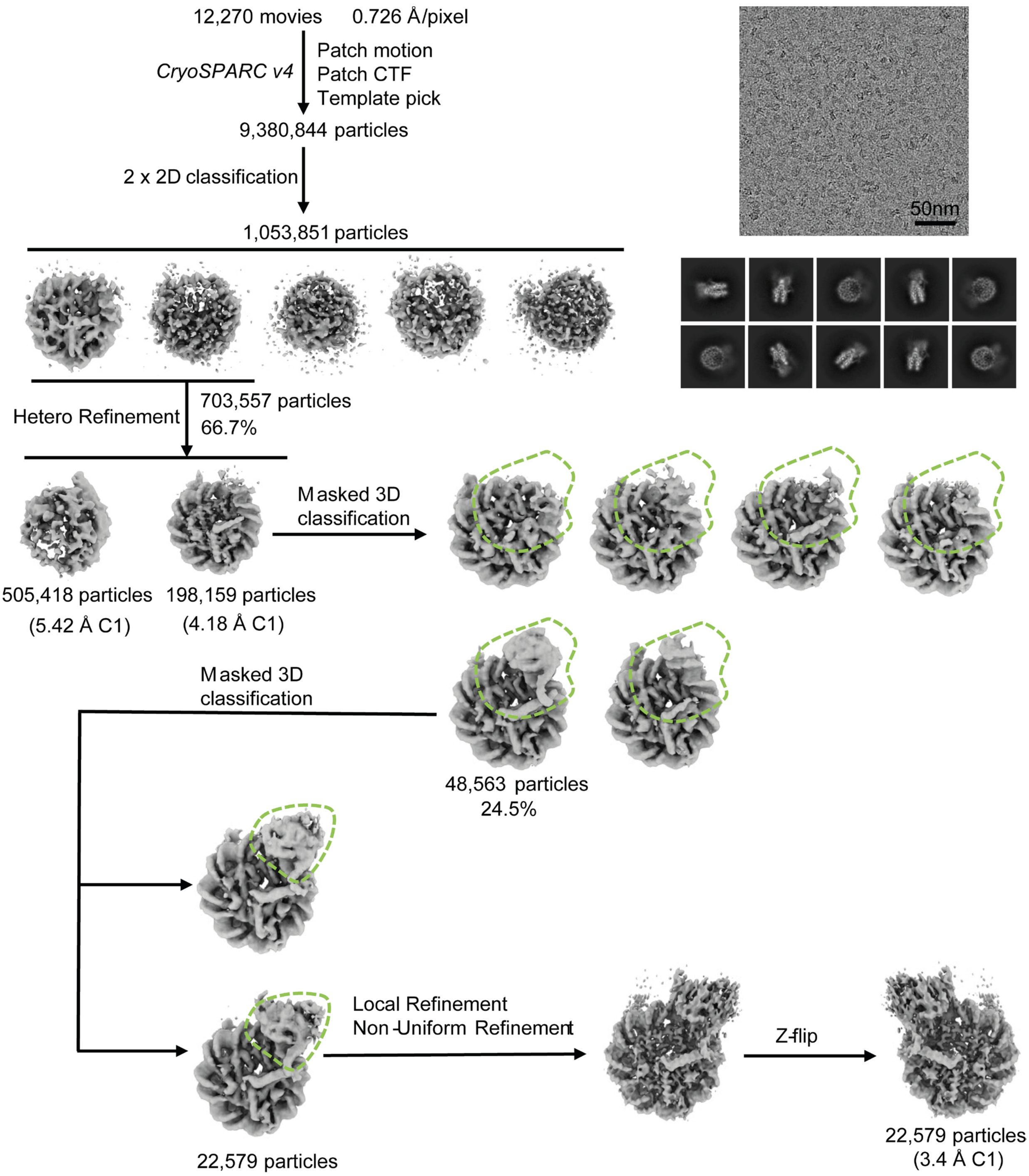

Supplementary Fig. 9. SIRT7:H3K18DTU complex data processing workflow. Processing workflow for SIRT7:H3K18DTU complex showing 2D class averages, 3D classification and refinement steps. Focused classification with the regions covered by the mask are also shown.

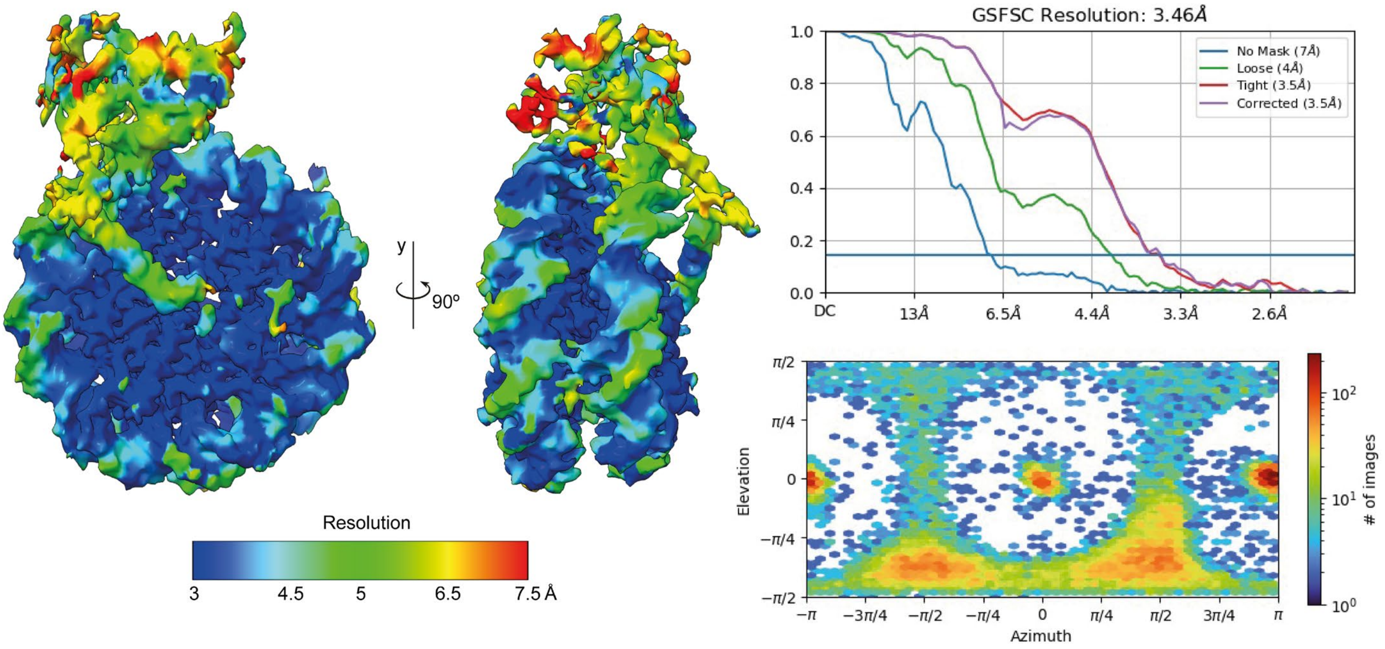

Supplementary Fig. 10. SIRT7:H3K18DTU cryo-EM. Local resolution map, Fourier Shell Correlation (FSC) curve and particle distribution orientation of the SIRT7:H3K18DTU-nucleosome complex.

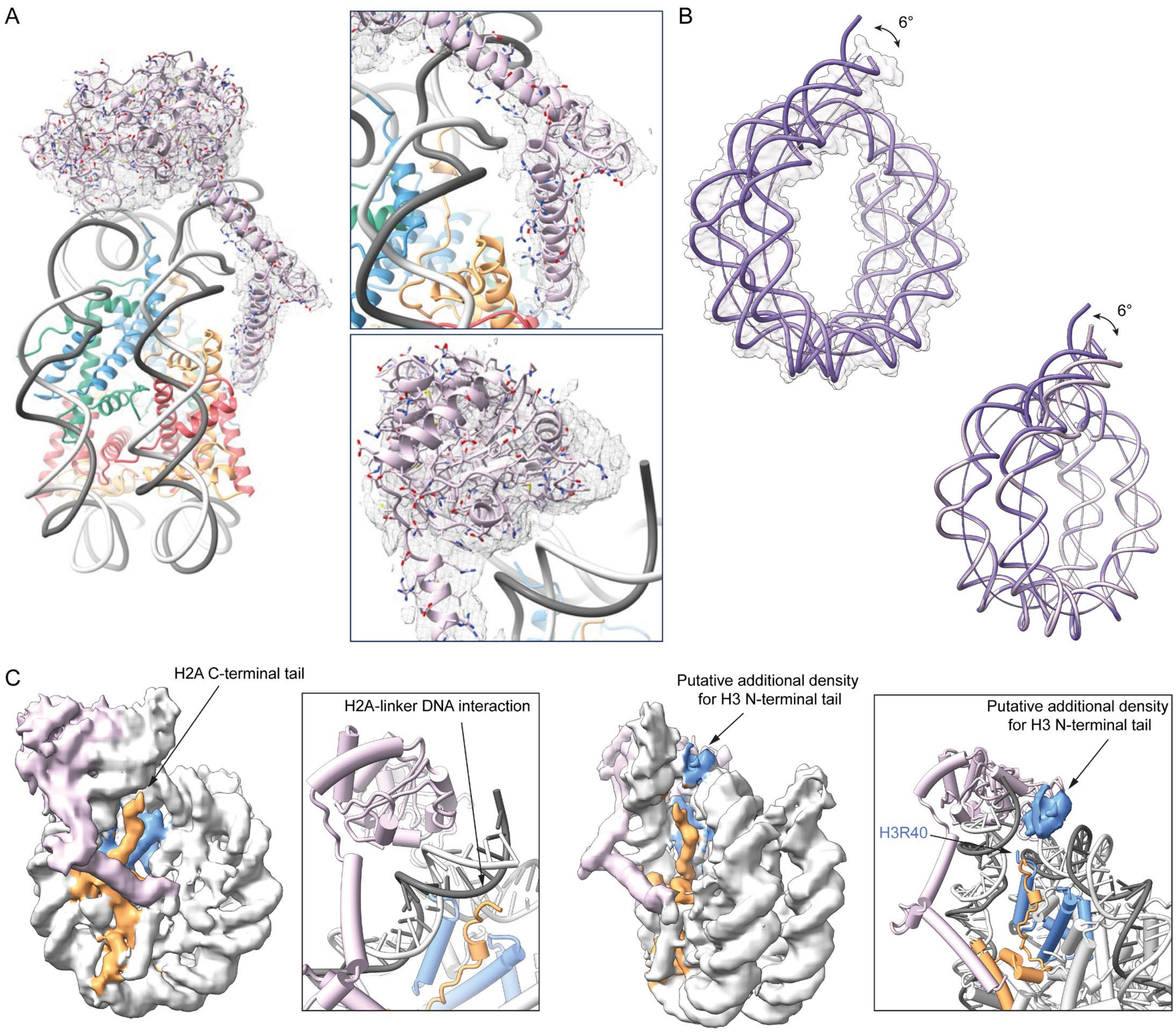

Supplementary Fig. 11. SIRT7:H3K18DTU model quality and structural details. A, Overall and zoom-in views of the cryo-EM map of structural elements of SIRT7 in the cryo-EM map (mesh representation) of the SIRT7:H3K18DTU-nucleosome complex. **B**, Measurement of the relative bending of the linker DNA axes, between the H3K36- (purple) and H3K18 (light pink)-bound structures. The H3K18-bound model is shown either as surface (top) or cartoon (bottom). **C**, H2A C-terminal and H3 N-terminal densities suggesting histone:DNA interactions that may further explain the relative bending of linker DNA.

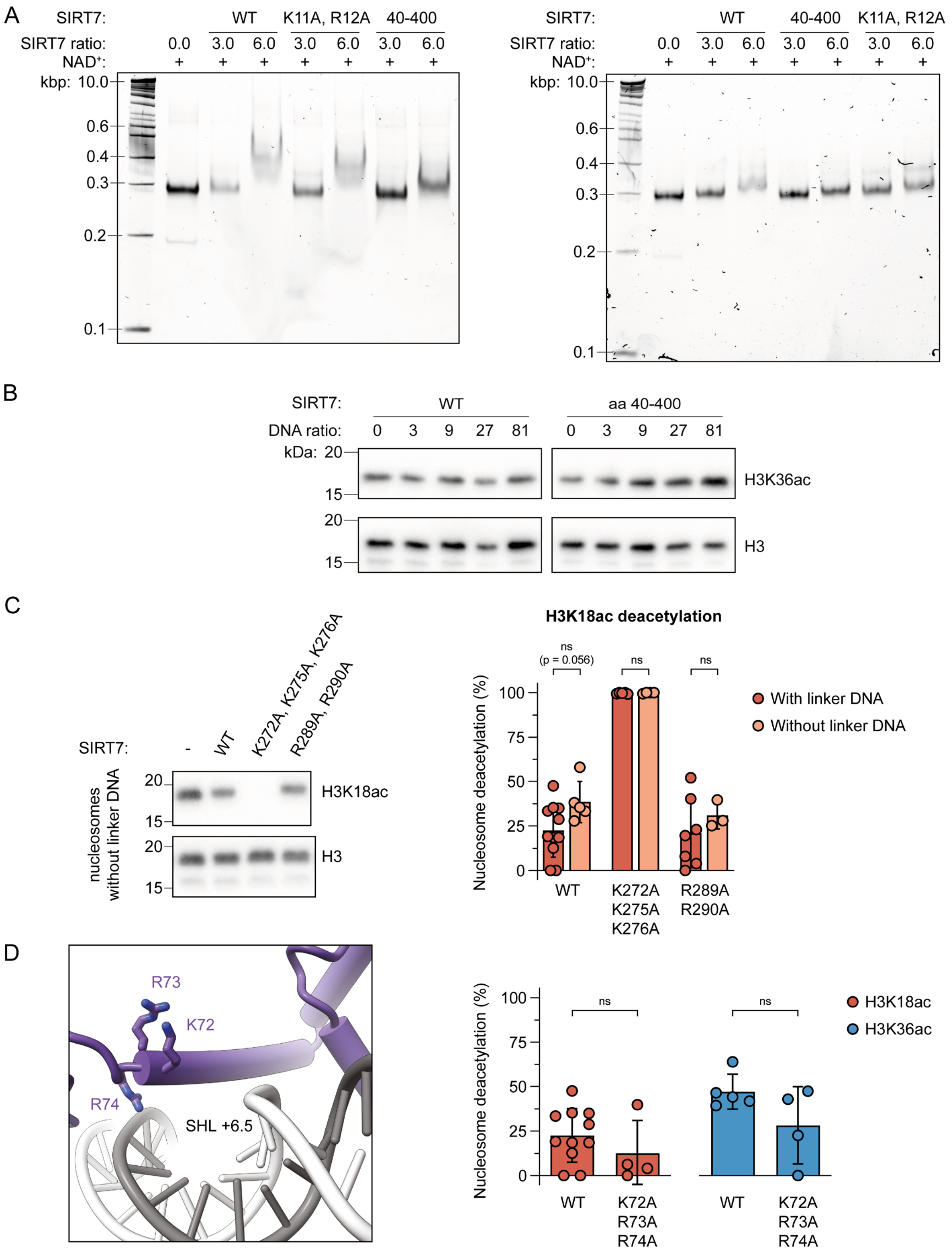

Supplementary Fig. 12. Additional data on SIRT7 mutant activity. A, EMSA replicates of unmodified nucleosomes (187 bp DNA) with wild type SIRT7, truncated SIRT7 (aa 40-400) and the K11A, R12A mutant. **B**, Inhibitory effect of free DNA (187 bp) on SIRT7 activity, measured by western blot. **C**, Activity of wild type SIRT7 and selected mutants on H3K18ac-modified nucleosomes with standard DNA (187 bp) and 601 DNA without linker ends (147 bp). Error bars represent mean ± SD (*n* = 3–11, as shown) of distinct samples. Statistical analysis: unpaired t tests, two-tailed, ns: $p > 0.05$. **D**, Detailed interactions of the third N-terminal helix (SHL: superhelical location) and quantification of nucleosome deacetylation normalized to H3 loading, at a concentration of wild type SIRT7 or the K72A, R73A and R74A mutant of 50 nM for H3K18ac and 3 nM for H3K36ac experiments. These mutations were observed to further promote the activation by 5S RNA[3]. Nucleosome concentration: 200 nM. Error bars represent mean ± SD (*n* = 4–11, as shown) of distinct samples. Statistical analysis: unpaired t tests, two-tailed, ns: $p > 0.05$. Source data are provided as a Source Data file.

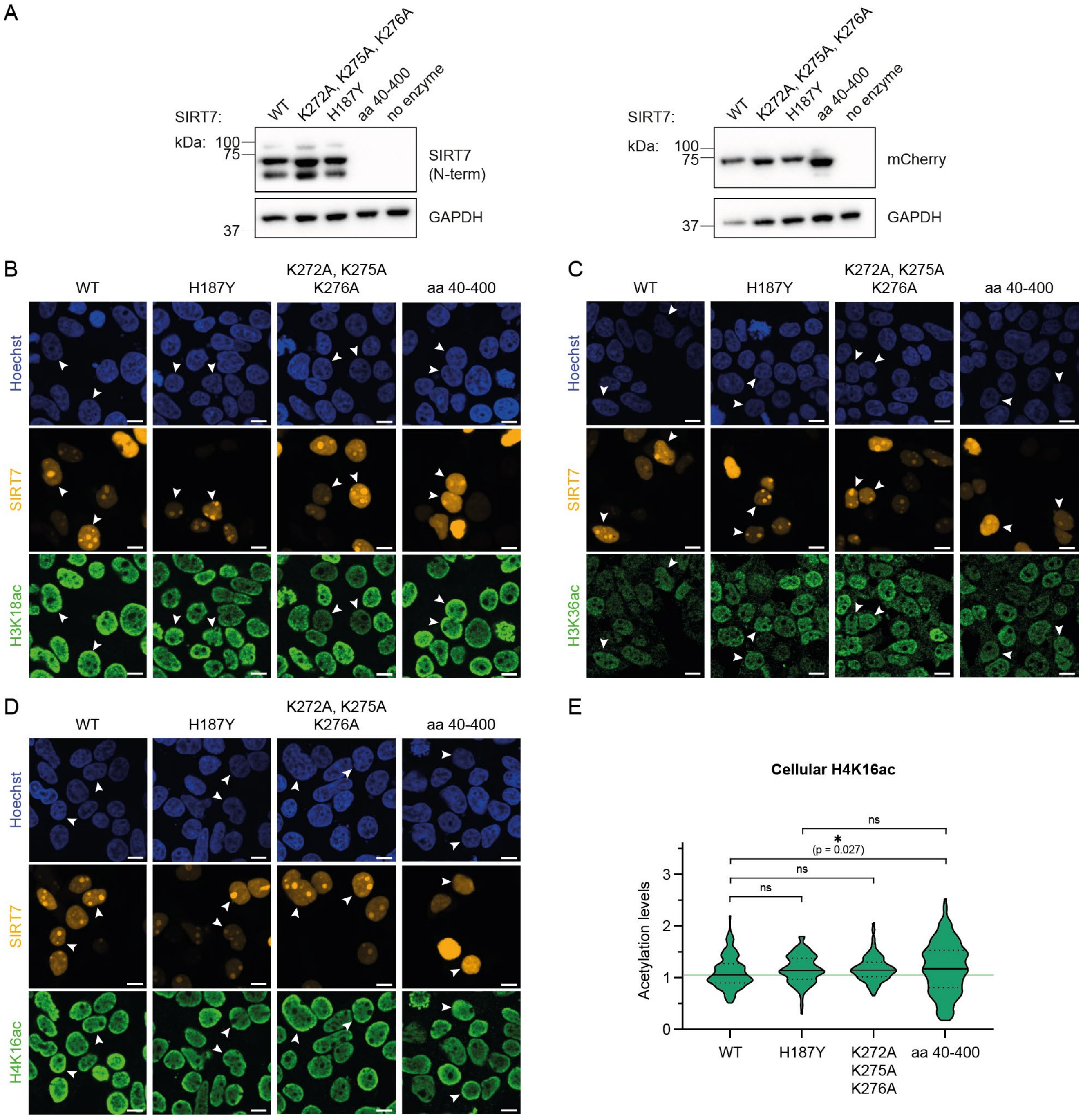

Supplementary Fig. 13. Substrate selectivity of SIRT7 mutants in cells. A, Western blot of HEK293F *SIRT7*$^{-/-}$ lysates upon transfection and incubation with SIRT7-mCherry constructs as indicated. Since the commercial SIRT7 antibody binds an N-terminal peptide that is missing in SIRT7(40–400), an mCherry antibody was used to detect this construct instead. **B**, Spinning-disk confocal images of HEK293F *SIRT7*$^{-/-}$ cells upon transient transfection and H3K18ac immunofluorescence. Scale bars represent 10 µm. **C**, Images for H3K36ac immunofluorescence. **D**, Images for H4K16ac immunofluorescence, used here as control. **E**, Violin plot (truncated) of H4K16ac levels in transfected cells. Lines represent median and quartile values of $n \geq 3$ distinct experiments (ns: $p > 0.05$) and light background line indicates median value for WT SIRT7. Source data are provided as a Source Data file.

Supplementary Tables 1–5

Supplementary Table 1. Recombinant SIRT7 constructs, primer sequences and HRMS analyses. Red amino acids: removed upon TEV protease cleavage. Bold underlined amino acids: mutations.

SIRT7 construct	Amino acid sequence	Primers used	HRMS
6xH-SIRT7	MRGSHHHHHH GMASMTGGQQ MGDLYDDDDK DHPFTENLYF QGMAAGGLSR SERKAAERVR RLREEQQRER LRQVSRILRK AAAERSAEEG RLLAESADLV TELQGRSRRR EGLKRRQEEV CDDPEELRGK VRELASAVRN AKYLVVYTGA GISTAASIPD YRGPNGVWTL LQKGRSVSAA DLSEAEPTLT HMSITRLHEQ KLVQHVVSQN CDGLHLRSGL PRTAISELHG NMYIEVCTSC VPNREYVRVF DVTERTALHR HQTGRTCHKC GTQLRDTIVH FGERGTLGQP LNWEAATEAA SRADTILCLG SSLKVLKKYP RLWCMTKPPS RRPKLYIVNL QWTPKDDWAA LKLHGKCDDV MRLLMAELGL EIPAYSRWQD PIFSLATPLR AGEEGSHSRK SLCRSREEAP PGDRGAPLSS APILGGWFGR GCTKRTKRKK VT	N/A	N/A
6xH-SIRT7 R11A,K12A	MRGSHHHHHH GMASMTGGQQ MGDLYDDDDK DHPFTENLYF QGMAAGGLSR SE**<u>AA</u>**AAERVR RLREEQQRER LRQVSRILRK AAAERSAEEG RLLAESADLV TELQGRSRRR EGLKRRQEEV CDDPEELRGK VRELASAVRN AKYLVVYTGA GISTAASIPD YRGPNGVWTL LQKGRSVSAA DLSEAEPTLT HMSITRLHEQ KLVQHVVSQN CDGLHLRSGL PRTAISELHG NMYIEVCTSC VPNREYVRVF DVTERTALHR HQTGRTCHKC GTQLRDTIVH FGERGTLGQP LNWEAATEAA SRADTILCLG SSLKVLKKYP RLWCMTKPPS RRPKLYIVNL QWTPKDDWAA LKLHGKCDDV MRLLMAELGL EIPAYSRWQD PIFSLATPLR AGEEGSHSRK SLCRSREEAP PGDRGAPLSS APILGGWFGR GCTKRTKRKK VT	F: 5'-CCGTAGCGAAGCGGCAGCAGCAGAACGTG TTCGTCG<br><br>R: 5'-CTCAGACCACCGGCTGC	calculated MW: 44813.2<br><br>found: 44812.6
6xH-SIRT7 K72A,R73A,R74A	MRGSHHHHHH GMASMTGGQQ MGDLYDDDDK DHPFTENLYF QGMAAGGLSR SERKAAERVR RLREEQQRER LRQVSRILRK AAAERSAEEG RLLAESADLV TELQGRSRRR EGL**<u>AAA</u>**QEEV CDDPEELRGK VRELASAVRN AKYLVVYTGA GISTAASIPD YRGPNGVWTL LQKGRSVSAA DLSEAEPTLT HMSITRLHEQ KLVQHVVSQN CDGLHLRSGL PRTAISELHG NMYIEVCTSC VPNREYVRVF DVTERTALHR HQTGRTCHKC GTQLRDTIVH FGERGTLGQP LNWEAATEAA SRADTILCLG SSLKVLKKYP RLWCMTKPPS RRPKLYIVNL QWTPKDDWAA LKLHGKCDDV MRLLMAELGL EIPAYSRWQD PIFSLATPLR AGEEGSHSRK SLCRSREEAP PGDRGAPLSS APILGGWFGR GCTKRTKRKK VT	F: 5'-GCGGCGGCGCAAGAAGAAGTTTGCGACGA TCCGG<br><br>R: 5'-CAGACCTTCACGACGACGG	calculated MW: 44728.1<br><br>found: 44728.2
6xH-SIRT7 H187Y	MRGSHHHHHH GMASMTGGQQ MGDLYDDDDK DHPFTENLYF QGMAAGGLSR SERKAAERVR RLREEQQRER LRQVSRILRK AAAERSAEEG RLLAESADLV TELQGRSRRR EGLKRRQEEV CDDPEELRGK VRELASAVRN AKYLVVYTGA GISTAASIPD YRGPNGVWTL LQKGRSVSAA DLSEAEPTLT HMSITRLHEQ KLVQHVVSQN CDGLHLRSGL PRTAISEL**<u>Y</u>**G NMYIEVCTSC VPNREYVRVF DVTERTALHR HQTGRTCHKC GTQLRDTIVH FGERGTLGQP LNWEAATEAA SRADTILCLG SSLKVLKKYP RLWCMTKPPS RRPKLYIVNL QWTPKDDWAA LKLHGKCDDV MRLLMAELGL EIPAYSRWQD PIFSLATPLR AGEEGSHSRK SLCRSREEAP PGDRGAPLSS APILGGWFGR GCTKRTKRKK VT	F: 5'-CGCAATTAGCGAACTGTATGGCAATATGT ATATTGAAG<br><br>R: 5'-GTACGAGGCAGACCGCTACG	calculated MW: 44981.5<br><br>found: 44981.4

6xH-SIRT7 H217A,R218A	MRGSHHHHHH GMASMTGGQQ MGDLYDDDDK DHPFTENLYF QGMAAGGLSR SERKAAERVR RLREEQQRER LRQVSRILRK AAAERSAEEG RLLAESADLV TELQGRSRRR EGLKRRQEEV CDDPEELRGK VRELASAVRN AKYLVVYTGA GISTAASIPD YRGPNGVWTL LQKGRSVSAA DLSEAEPTLT HMSITRLHEQ KLVQHVVSQN CDGLHLRSGL PRTAISELHG NMYIEVCTSC VPNREYVRVF DVTERTAL**AA** HQTGRTCHKC GTQLRDTIVH FGERGTLGQP LNWEAATEAA SRADTILCLG SSLKVLKKYP RLWCMTKPPS RRPKLYIVNL QWTPKDDWAA LKLHGKCDDV MRLLMAELGL EIPAYSRWQD PIFSLATPLR AGEEGSHSRK SLCRSREEAP PGDRGAPLSS APILGGWFGR GCTKRTKRKK VT	F: 5’-GAACGCACCGCACTGGCGGCGCATCAGA CCGGTCGTACCTG<br><br>R: 5’-GGTCACATCAAAAACACGAACATATTCAC GAT	calculated MW: 44804.3<br><br>found: 44803.0
6xH-SIRT7 K272A,K275A, K276A	MRGSHHHHHH GMASMTGGQQ MGDLYDDDDK DHPFTENLYF QGMAAGGLSR SERKAAERVR RLREEQQRER LRQVSRILRK AAAERSAEEG RLLAESADLV TELQGRSRRR EGLKRRQEEV CDDPEELRGK VRELASAVRN AKYLVVYTGA GISTAASIPD YRGPNGVWTL LQKGRSVSAA DLSEAEPTLT HMSITRLHEQ KLVQHVVSQN CDGLHLRSGL PRTAISELHG NMYIEVCTSC VPNREYVRVF DVTERTALHR HQTGRTCHKC GTQLRDTIVH FGERGTLGQP LNWEAATEAA SRADTILCLG SSL**A**VL**AA**YP RLWCMTKPPS RRPKLYIVNL QWTPKDDWAA LKLHGKCDDV MRLLMAELGL EIPAYSRWQD PIFSLATPLR AGEEGSHSRK SLCRSREEAP PGDRGAPLSS APILGGWFGR GCTKRTKRKK VT	F: 5’-CGCACCGCACTGGCGGCGCATCAGACCG GTCGTACC<br><br>R: 5’-TTCGGTCACATCAAAAACACGAACATATTC AC	calculated MW: 44784.2<br><br>found: 44783.8
6xH-SIRT7 R289A,R290A	MRGSHHHHHH GMASMTGGQQ MGDLYDDDDK DHPFTENLYF QGMAAGGLSR SERKAAERVR RLREEQQRER LRQVSRILRK AAAERSAEEG RLLAESADLV TELQGRSRRR EGLKRRQEEV CDDPEELRGK VRELASAVRN AKYLVVYTGA GISTAASIPD YRGPNGVWTL LQKGRSVSAA DLSEAEPTLT HMSITRLHEQ KLVQHVVSQN CDGLHLRSGL PRTAISELHG NMYIEVCTSC VPNREYVRVF DVTERTALHR HQTGRTCHKC GTQLRDTIVH FGERGTLGQP LNWEAATEAA SRADTILCLG SSLKVLKKYP RLWCMTKPPS **AA**PKLYIVNL QWTPKDDWAA LKLHGKCDDV MRLLMAELGL EIPAYSRWQD PIFSLATPLR AGEEGSHSRK SLCRSREEAP PGDRGAPLSS APILGGWFGR GCTKRTKRKK VT	F: 5’-GTGTATGACCAAACCTCCGAGTGCGGCT CCGAAACTGTATATTGTTAATCTGCAGTG<br><br>R: 5’-CACAGACGCGGATACTTTTTCAGAAC	calculated MW: 44785.2<br><br>found: 44784.4
6xH-SIRT7 (40-400)	MRGSHHHHHH GMASMTGGQQ MGDLYDDDDK DHPFTENLYF QGMAAERSAE EGRLLAESAD LVTELQGRSR RREGLKRRQE EVCDDPEELR GKVRELASAV RNAKYLVVYT GAGISTAASI PDYRGPNGVW TLLQKGRSVS AADLSEAEPT LTHMSITRLH EQKLVQHVVS QNCDGLHLRS GLPRTAISEL HGNMYIEVCT SCVPNREYVR VFDVTERTAL HRHQTGRTCH KCGTQLRDTI VHFGERGTLG QPLNWEAATE AASRADTILC LGSSLKVLKK YPRLWCMTKP PSRRPKLYIV NLQWTPKDDW AALKLHGKCD DVMRLLMAEL GLEIPAYSRW QDPIFSLATP LRAGEEGSHS RKSLCRSREE APPGDRGAPL SSAPILGGWF GRGCTKRTKR KKVT	F: 5’-GCAGCCGAACGTAGCGC<br><br>R: 5’-CATACCTTGAAAATACAGGTTCTCGGTG	calculated MW: 40456.3<br><br>found: 40455.0

Supplementary Table 2. Nucleosome-positioning DNAs and primers. Red: flanking DNA.

Sample	Nucleotide sequence (double-stranded DNA)	Primers used	Calc. extinction coefficient (260 nm, $M^{-1} \cdot cm^{-1}$)
147 bp DNA	5’-CTGGAGAATCCCGGTGCCGAGGCCGCTCAAT TGGTCGTAGACAGCTCTAGCACCGCTTAAACGC ACGTACGCGCTGTCCCCCGCGTTTTAACCGCCA AGGGGATTACTCCCTAGTCTCCAGGCACGTGTC AGATATATACAAGATCC	F: 5’-CTGGAGAATCCCGGTGCC<br><br>R: 5’-GGATCTTGTATATATCTGACACGTGCCTG	2426422
187 bp DNA	5’-GTAATGTAATGTAATGTAATCTGGAGAATCCC GGTGCCGAGGCCGCTCAATTGGTCGTAGACAG CTCTAGCACCGCTTAAACGCACGTACGCGCTG TCCCCCGCGTTTTAACCGCCAAGGGGATTACT CCCTAGTCTCCAGGCACGTGTCAGATATATAC AAGATCCCCTTACCTTACCTTACCTTA	F: 5’-GTAATGTAATGTAATGTAATCTGGAGAATC CCGGTGCC<br><br>R: 5’-TAAGGTAAGGTAAGGTAAGGGGATC TTGTATATATCTGACACGTGC	3072083

Supplementary Table 3. Cryo-EM data collection, refinement and validation statistics for SIRT7-nucleosome complexes.

Data collection and processing	SIRT7:H3K36MTU nucleosome complex<br>(EMD-51453)<br>(PDB 9GMR)	SIRT7:H3K18DTU nucleosome complex<br>(EMD-51449)<br>(PDB 9GMK)
Electron Microscope	Titan Krios G4 (ColdFEG, Falcon4i)	Titan Krios G4 (ColdFEG, Falcon4i, SelectrisX)
Nominal Magnification	96kx	165kx
Voltage (kV)	300	300
Recorded Micrographs	8 623	12 270
Electron exposure (e–/Å2)	50	50
Defocus range (μm)	0.8-2.5	0.8-2.5
Pixel size (Å)	0.82	0.726
Symmetry imposed	C1	C1
Initial particle images (no.)	7 266 736	9 149 090
Final particle images (no.)	337 476	22 579
Map resolution (Å)	2.8	3.5
FSC threshold	0.143	0.143
Map resolution range (Å)	5.0-2.0	7.0-3.0
Map sharpening B factor (Å2)	-76.1	-42.9
**Refinement**		
Initial model used (PDB code)	3LZ0	3LZ0
Model composition<br>Atoms (hydrogens)<br>Protein residues<br>Nucleotide residues<br>Ligands	14765 (0)<br>1086<br>298<br>LIG: 1<br>ZN: 1	14643 (0)<br>1081<br>296
B factors ($Å^2$)<br>Protein<br>Nucleotide<br>Ligand	0.00/90.00/38.52<br>16.97/159.14/73.62<br>59.82/100.72/67.23	232.91/799.07/391.79<br>257.47/587.40/348.43
R.m.s. deviations<br>Bond lengths (Å)<br>Bond angles (°)	0.004 (0)<br>0.539 (0)	0.004 (0)<br>0.739 (31)
Validation<br>MolProbity score<br>Clashscore<br>Poor rotamers (%)	1.88<br>6.09<br>3.09	1.83<br>21.52<br>0.33
Ramachandran plot<br>Favored (%)<br>Allowed (%)<br>Disallowed (%)	97.00<br>3.00<br>0.00	98.02<br>1.98<br>0.00

Supplementary Table 4. SIRT7 constructs for mammalian cell expression and primer sequences. Red amino acids: mCherry sequence. Bold underlined amino acids: mutations.

SIRT7 construct	Amino acid sequence	Primers used
SIRT7-mCherry-HA	MAAGGLSRSE RKAAERVRRL REEQRERLR QVSRILRKAA<br>AERSAEEGRL LAESADLVTE LQGRSRRREG LKRRQEEVCD<br>DPEELRGKVR ELASAVRNAK YLVVYTGAGI STAASIPDYR<br>GPNGVWTLLQ KGRSVSAADL SEAEPTLTHM SITRLHEQKL<br>VQHVVSQNCD GLHLRSGLPR TAISELHGNM YIEVCTSCVP<br>NREYVRVFDV TERTALHRHQ TGRTCHKCGT QLRDTIVHFG<br>ERGTLGQPLN WEAATEAASR ADTILCLGSS LKVLKKYPRL<br>WCMTKPPSRR PKLYIVNLQW TPKDDWAALK LHGKCDDVMR<br>LLMAELGLEI PAYSRWQDPI FSLATPLRAG EEGSHSRKSL<br>CRSREEAPPG DRGAPLSSAP ILGGWFGRGC TKRTKRKKVT<br>AGGGGSGGGG SGGGGSVSKG EEDNMAIIKE FMRFKVHMEG<br>SVNGHEFEIE GEGEGRPYEG TQTAKLKVTK GGPLPFAWDI<br>LSPQFMYGSK AYVKHPADIP DYLKLSFPEG FKWERVMNFE<br>DGGVVTVTQD SSLQDGEFIY KVKLRGTNFP SDGPVMQKKT<br>MGWEASSERM YPEDGALKGE IKQRLKLKDG GHYDAEVKTT<br>YKAKKPVQLP GAYNVNIKLD ITSHNEDYTI VEQYERAEGR<br>HSTGGMDELY KGSGYPYDVP DYA	N/A
SIRT7(H187Y)-mCherry-HA	MAAGGLSRSE RKAAERVRRL REEQRERLR QVSRILRKAA<br>AERSAEEGRL LAESADLVTE LQGRSRRREG LKRRQEEVCD<br>DPEELRGKVR ELASAVRNAK YLVVYTGAGI STAASIPDYR<br>GPNGVWTLLQ KGRSVSAADL SEAEPTLTHM SITRLHEQKL<br>VQHVVSQNCD GLHLRSGLPR TAISEL**Y**GNM YIEVCTSCVP<br>NREYVRVFDV TERTALHRHQ TGRTCHKCGT QLRDTIVHFG<br>ERGTLGQPLN WEAATEAASR ADTILCLGSS LKVLKKYPRL<br>WCMTKPPSRR PKLYIVNLQW TPKDDWAALK LHGKCDDVMR<br>LLMAELGLEI PAYSRWQDPI FSLATPLRAG EEGSHSRKSL<br>CRSREEAPPG DRGAPLSSAP ILGGWFGRGC TKRTKRKKVT<br>AGGGGSGGGG SGGGGSVSKG EEDNMAIIKE FMRFKVHMEG<br>SVNGHEFEIE GEGEGRPYEG TQTAKLKVTK GGPLPFAWDI<br>LSPQFMYGSK AYVKHPADIP DYLKLSFPEG FKWERVMNFE<br>DGGVVTVTQD SSLQDGEFIY KVKLRGTNFP SDGPVMQKKT<br>MGWEASSERM YPEDGALKGE IKQRLKLKDG GHYDAEVKTT<br>YKAKKPVQLP GAYNVNIKLD ITSHNEDYTI VEQYERAEGR<br>HSTGGMDELY KGSGYPYDVP DYA	F: 5'-GCCATCTCCGAGCTCTACGGGAACATGTA CATTGAAGTC<br><br>R: 5'-CGTGCGCGGCAGCCCACTCCTCAGG
SIRT7(K272A,K275A, K276A)-mCherry-HA	MAAGGLSRSE RKAAERVRRL REEQRERLR QVSRILRKAA<br>AERSAEEGRL LAESADLVTE LQGRSRRREG LKRRQEEVCD<br>DPEELRGKVR ELASAVRNAK YLVVYTGAGI STAASIPDYR<br>GPNGVWTLLQ KGRSVSAADL SEAEPTLTHM SITRLHEQKL<br>VQHVVSQNCD GLHLRSGLPR TAISELHGNM YIEVCTSCVP<br>NREYVRVFDV TERTALHRHQ TGRTCHKCGT QLRDTIVHFG<br>ERGTLGQPLN WEAATEAASR ADTILCLGSS L**A**VL**AA**YPRL<br>WCMTKPPSRR PKLYIVNLQW TPKDDWAALK LHGKCDDVMR<br>LLMAELGLEI PAYSRWQDPI FSLATPLRAG EEGSHSRKSL<br>CRSREEAPPG DRGAPLSSAP ILGGWFGRGC TKRTKRKKVT<br>AGGGGSGGGG SGGGGSVSKG EEDNMAIIKE FMRFKVHMEG<br>SVNGHEFEIE GEGEGRPYEG TQTAKLKVTK GGPLPFAWDI<br>LSPQFMYGSK AYVKHPADIP DYLKLSFPEG FKWERVMNFE<br>DGGVVTVTQD SSLQDGEFIY KVKLRGTNFP SDGPVMQKKT<br>MGWEASSERM YPEDGALKGE IKQRLKLKDG GHYDAEVKTT<br>YKAKKPVQLP GAYNVNIKLD ITSHNEDYTI VEQYERAEGR<br>HSTGGMDELY KGSGYPYDVP DYA	F: 5'-GCGGTTCTAGCCGCCTACCCACGCCTCTG GTGC<br><br>R: 5'-CAGGCTGGACCCTAGACACAG
SIRT7(40-400)-mCherry-HA	MAAERSAEEG RLLAESADLV TELQGRSRRR EGLKRRQEEV<br>CDDPEELRGK VRELASAVRN AKYLVVYTGA GISTAASIPD<br>YRGPNGVWTL LQKGRSVSAA DLSEAEPTLT HMSITRLHEQ<br>KLVQHVVSQN CDGLHLRSGL PRTAISELHG NMYIEVCTSC<br>VPNREYVRVF DVTERTALHR HQTGRTCHKC GTQLRDTIVH<br>FGERGTLGQP LNWEAATEAA SRADTILCLG SSLKVLKKYP<br>RLWCMTKPPS RRPKLYIVNL QWTPKDDWAA LKLHGKCDDV<br>MRLLMAELGL EIPAYSRWQD PIFSLATPLR AGEEGSHSRK<br>SLCRSREEAP PGDRGAPLSS APILGGWFGR GCTKRTKRKK<br>VTAGGGGSGG GGSGGGGSVS KGEEDNMAII KEFMRFKVHM<br>EGSVNGHEFE IEGEGEGRPY EGTQTAKLKV TKGGPLPFAW<br>DILSPQFMYG SKAYVKHPAD IPDYLKLSFP EGFKWERVMN<br>FEDGGVVTVT QDSSLQDGEF IYKVKLRGTN FPSDGPVMQK<br>KTMGWEASSE RMYPEDGALK GEIKQRLKLK DGGHYDAEVK<br>TTYKAKKPVQ LPGAYNVNIK LDITSHNEDY TIVEQYERAE<br>GRHSTGGMDE LYKGSGYPYD VPDYA	F: 5'-GAGCGCAGCGCCGAGGAGGGC<br><br>R: 5'-GGCTGCCATCGCTCCCCTGGAGAAG

Supplementary Table 5. Recombinant histone constructs. Red amino acids: removed upon Ulp1 treatment. Bold underlined: native chemical ligation site.

Histone construct	Amino acid sequence
6xH-SUMO-H3(29-135) A29C, C110A	MGSSHHHHHH GSGLVPRGSA SMSDSEVNQE AKPEVKPEVK PETHINLKVS DGSSEIFFKI KKTTPLRRLM EAFAKRQGKE MDSLRFLYDG IRIQADQTPE DLDMEDNDII EAHREQIGG**C** PATGGVKKPH RYRPGTVALR EIRRYQKSTE LLIRKLPFQR LVREIAQDFK TDLRFQSSAV MALQEASEAY LVGLFEDTNL AAIHAKRVTI MPKDIQLARR IRGERA
6xH-SUMO-H3(47-135) A47C, C110A	MGSSHHHHHH GSGLVPRGSA SMSDSEVNQE AKPEVKPEVK PETHINLKVS DGSSEIFFKI KKTTPLRRLM EAFAKRQGKE MDSLRFLYDG IRIQADQTPE DLDMEDNDII EAHREQIGG**C** LREIRRYQKS TELLIRKLPF QRLVREIAQD FKTDLRFQSS AVMALQEASE AYLVGLFEDT NLAAIHAKRV TIMPKDIQLA RRIRGERA
H. sapiens H2A type 2-A	SGRGKQGGKA RAKAKSRSSR AGLQFPVGRV HRLLRKGNYA ERVGAGAPVY MAAVLEYLTA EILELAGNAA RDNKKTRIIP RHLQLAIRND EELNKLLGKV TIAQGGVLPN IQAVLLPKKT ESHHKAKGK
H. sapiens H2B type 1-K	PEPAKSAPAP KKGSKKAVTK AQKKDGKKRK RSRKESYSVY VYKVLKQVHP DTGISSKAMG IMNSFVNDIF ERIAGEASRL AHYNKRSTIT SREIQTAVRL LLPGELAKHA VSEGTKAVTK YTSAK
H. sapiens H3.2 with C110A mutation	ARTKQTARKS TGGKAPRKQL ATKAARKSAP ATGGVKKPHR YRPGTVALRE IRRYQKSTEL LIRKLPFQRL VREIAQDFKT DLRFQSSAVM ALQEASEAYL VGLFEDTNLA AIHAKRVTIM PKDIQLARRI RGERA
H. sapiens H4	SGRGKGGKGL GKGGAKRHRK VLRDNIQGIT KPAIRRLARR GGVKRISGLI YEETRGVLKV FLENVIRDAV TYTEHAKRKT VTAMDVVYAL KRQGRTLYGF GG

Chemical synthesis

General methods

All commercial reagents and solvents were of analytical grade and used without further purification. H_2O was of MilliQ grade unless otherwise stated and obtained from a Thermo Scientific GenPure UF system. Reactions were monitored by HPLC-MS analysis using a Shimadzu MS2020 instrument equipped with a Waters Acquity UPLC C18 column (for peptide analysis) and a Waters Acquity UPLC C4 column (for protein analysis), and UV diode array and single quadrupole analysis systems. Gradients of eluent I (0.05% HCOOH in H_2O) and eluent II (0.05% HCOOH in MeCN) were used as mobile phase. Purification and chromatographic analyses were performed by reverse-phase HPLC on Agilent 1260 systems, with Zorbax 300SB-C18 columns #1 (7 µm, 250×21.2 mm, 300 Å, for preparative purification), #2 (5 µm, 250×9.4 mm, 300 Å, for semi-preparative purification), and #3 (5 µm, 150×4.60 mm, 300 Å, for analysis), using gradients of eluent III (0.1% TFA in H_2O) and eluent IV (0.1% TFA in MeCN/H_2O 90:10) at a flow rate of 20 mL/min (preparative purification), 4 mL/min (semi-preparative purification) or 1 mL/min (analysis). Identification of purification fractions and of the final products consisted on high-resolution MS analysis using a Waters Xevo G2-XS instrument equipped with UV diode array and quadrupole-time-of-flight analysis systems, by direct injection.

Peptide synthesis

Peptide synthesis was performed based on the protocol by Guidotti *et al.*, 2020[4].

To prepare hydrazide-modified resins, 2-chlorotrityl chloride resin (0.5 g, 1.55 mmol/g, Merck, cat. # 8.55017) was swelled in a round-bottom flask with DMF (3 mL) for 15 min at room temperature, followed by cooling in an ice bath and addition of a solution of hydrazine (1 mL, 1.65 M hydrazine monohydrate and 2.45 M *i*Pr_2EtN in DMF) dropwise under gentle stirring. The mixture was allowed to reach room temperature and further stirred for 1 h, after which MeOH (0.1 mL) was added and the mixture was stirred for additional 10 min. The resin was then transferred to vessels for manual solid phase peptide synthesis (SPPS), washed with DMF (3 × 2 min), and dried under suction. A solution of Fmoc-Val-OH or Fmoc-Ser(*t*Bu)-OH (2.5 equiv.), 1-[bis(dimethylamino)methylene]-1*H*-1,2,3-triazolo[4,5-*b*]pyridinium 3-oxid hexafluorophosphate (HATU, 2.38 equiv.) and *i*Pr_2EtN (5.0 equiv.) was prepared in DMF (for a 0.5 M concentration of HATU), incubated for 1 min at room temperature, and added to the dried resin. The reaction vessel was agitated gently for 1 h at room temperature, after which the resin was dried under suction and washed with DMF (3 × 2 min), CH_2Cl_2 (3 × 2 min) and MeOH (3 × 2 min), and dried under vacuum overnight. Loading of the resin was determined as reported[5]. Briefly, 1-3 mg resin aliquots were incubated with piperidine/DMF (4 mL, 20:80, v/v) for 30 min at room temperature, and the absorption of the solution at 301 nm was used to calculate the loading (0.6-0.7 mmol/g, Supplementary Fig. 14).

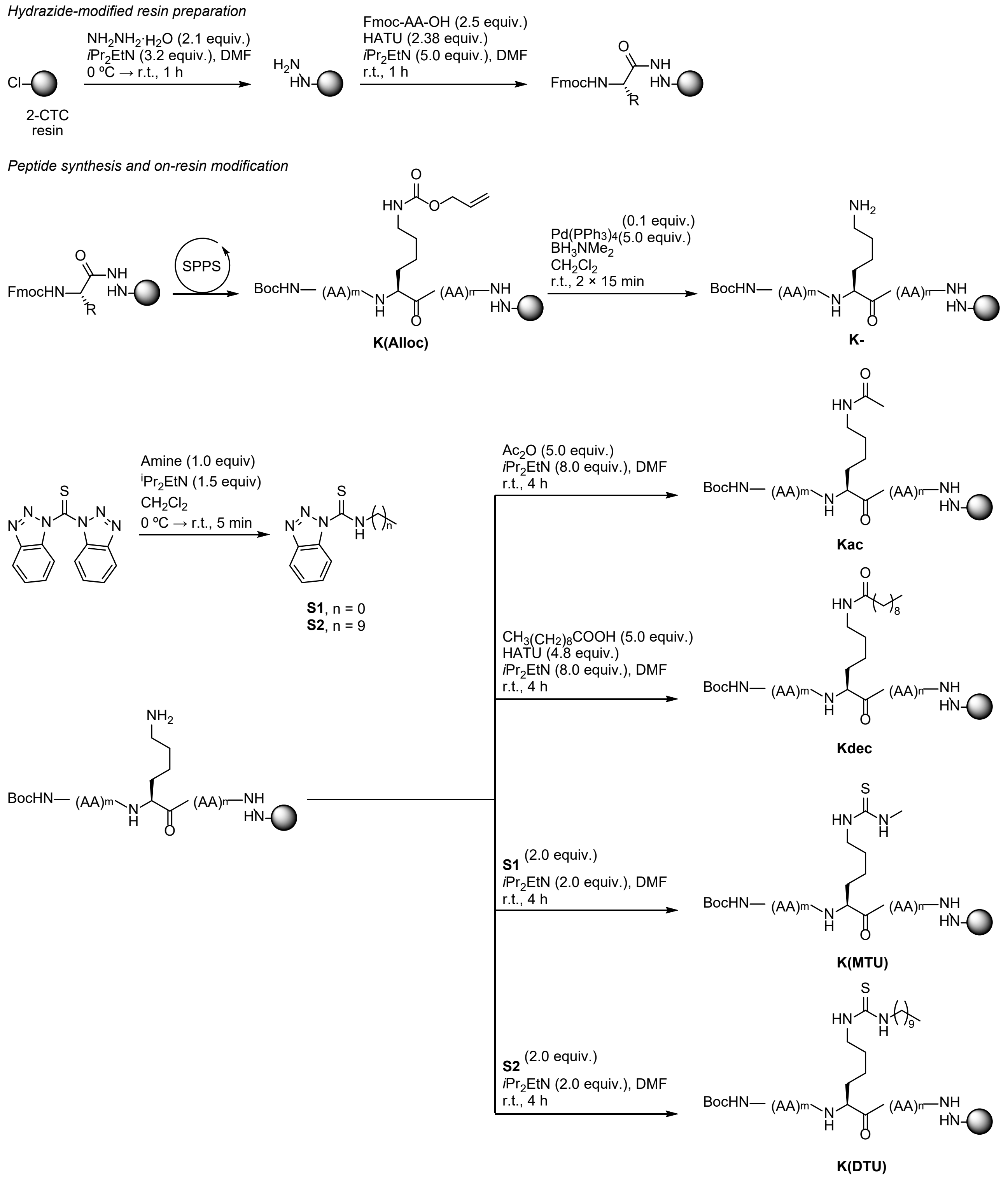

Supplementary Fig. 14. Summary of peptide synthesis and on-resin modification methods.

Standard Fmoc/t-Bu SPPS was performed on a Tribute peptide synthesizer (Gyros Protein Technologies, Sweden) using hydrazide-modified resins (0.05 or 0.1 mmol), *O*-(1*H*-6-chlorobenzotriazole-1-yl)-1,1,3,3-tetramethyluronium hexafluorophosphate (HCTU) as coupling reagent, DMF as solvent, and protected amino acids as follow: Boc-Ala-OH, Boc-Thz-OH, Fmoc-Ala-OH, Fmoc-Arg(Pbf)-OH, Fmoc-Gln(Trt)-OH, Fmoc-Gly-OH, Fmoc-

His(Trt)-OH, Fmoc-Leu-OH, Fmoc-Lys(Alloc)-OH, Fmoc-Lys(Boc)-OH, Fmoc-Pro-OH, Fmoc-Ser(*t*Bu)-OH, Fmoc-Thr(*t*Bu)-OH, Fmoc-Tyr(*t*Bu)-OH and Fmoc-Val-OH. The following pseudo-proline building blocks were used to improve synthetic yields: Fmoc-Ala-Thr[ψ(Me,Me)pro]-OH, Fmoc-Gln(Trt)-Thr[ψ(Me,Me)pro]-OH and Fmoc-Lys(Boc)-Ser[ψ(Me,Me)pro]-OH (see peptide sequences below). Fmoc deprotection was performed using piperidine/DMF (20:80, v/v) for 2 × 3 min. Amino acids (5.0 equiv.) were pre-activated with HCTU (4.8 equiv.) and *i*Pr_2EtN (7.0 equiv.) at a concentration of 0.25 M, added to the resin, and incubated by shaking for 30 min at room temperature. Reactions were repeated when indicated (double coupling, see peptide sequences below). Resin washes were performed with DMF. Resin aliquots were cleaved using a mixture of TFA/TIPS/H_2O (95:2.5:2.5) for reaction monitoring by HPLC-MS.

Peptide sequences prepared (underlined residues were double-coupled, and residues in italics correspond to pseudo-proline building blocks):

K18Alloc: Boc-A R T K *QT* A R *KS* T G G K A P R K(Alloc) Q L *AT* K A A R K S-NHNH-resin

K36Alloc: Boc-Thz P A T G G V K(Alloc) K P H R Y R P G T V-NHNH-resin

On-resin lysine modification

On-resin selective deprotection of K(Alloc) was performed in syringe reactors with a mixture $Pd(PPh_3)_4$ (0.1 equiv.) and BH_3NMe_2 (5.0 equiv.) in CH_2Cl_2 for 15 min at room temperature. The resins were then washed with CH_2Cl_2 (3 × 2 min) and the treatment was repeated. Resins were finally washed with MeOH (3 × 2 min) and dried under vacuum for storage, or used directly in the next step (Supplementary Fig. 14).

On-resin formation of Kac and Kdec was achieved by incubation of Alloc-deprotected peptidyl-resin samples (1.0 equiv.) with a solution of Ac_2O (5.0 equiv.) and *i*Pr_2EtN (8.0 equiv.), or decanoic acid (5.0 equiv.), HATU (4.8 equiv.) and *i*Pr_2EtN (8.0 equiv.), in DMF (at a concentration of acylating reagent of 0.1 M) for 4 h at room temperature, followed by washes with DMF (3 × 2 min), CH_2Cl_2 (3 × 2 min) and MeOH (3 × 2 min), and drying under vacuum.

On-resin thiourea formation was achieved as reported[6]. A solution of $MeNH_2$ (2 M in DMF, 2.0 equiv.) and *i*Pr_2EtN (3.0 equiv.), or n-decylamine (2.0 equiv.) and *i*Pr_2EtN (3.0 equiv.), in anhydrous CH_2Cl_2 (to a concentration of amine of 40 mM) was added dropwise to a solution of bis(benzotriazol-1-yl)methanethione (75 mM in anhydrous CH_2Cl_2, 2.0 equiv.) in an ice bath over 5 min. The resulting mixtures were concentrated under reduced pressure, diluted with a solution of *i*Pr_2EtN (75 mM in anhydrous DMF, 2.0 equiv.), and quickly added to the Alloc-deprotected peptidyl-resin (1.0 equiv.). The reactions were shaken for 4 h at room temperature, followed by washes with DMF (3 × 2 min), CH_2Cl_2 (3 × 2 min) and MeOH (3 × 2 min), and drying under vacuum.

All reactions were followed by test cleavage of peptidyl-resin aliquots using a mixture of TFA/TIPS/H_2O (95:2.5:2.5) and monitoring by HPLC-MS, and repeated when necessary.

Peptide cleavage and deprotection

Overall peptide deprotection and cleavage from resin was achieved by shaking of peptidyl-resin samples with a mixture of TFA/2,2′-(ethylenedioxy)diethanethiol (DODT)/TIPS/H_2O (92.5:2.5:2.5:2.5, 1 mL per 100 mg of resin) for 2-3 h at room temperature. The resulting solutions were concentrated under N_2 stream and triturated with ice-cold Et_2O. The mixtures were centrifuged (3000 g, 3 min, 4 °C), and the pellets were washed again with ice-cold Et_2O, separated by centrifugation (3000 g, 3 min, 4 °C), dried under N_2 stream and purified by preparative HPLC as described.

The following peptides were obtained (Supplementary Fig. 15 and Supplementary Table 6):

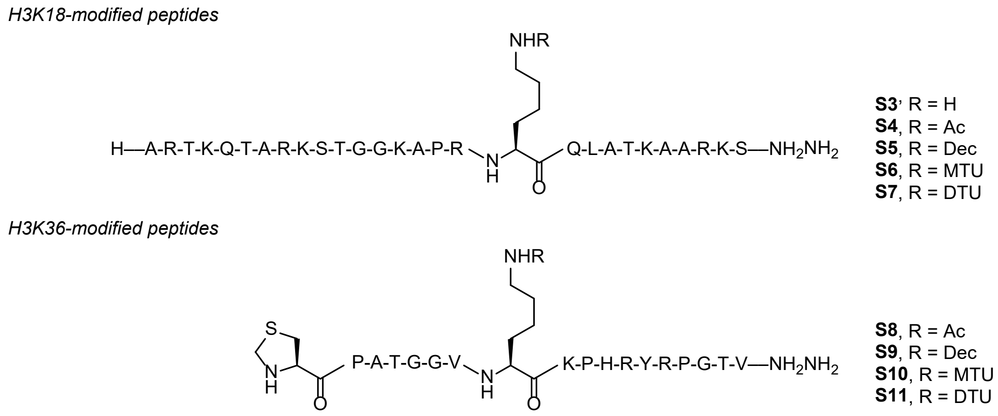

Supplementary Fig. 15. Chemical structure of synthesized peptides.

Supplementary Table 6. Peptide analyses and synthetic yields.

Peptide	Modification	Purity*	Formula	HRMS	Calcd. mass	Synth. yield
**S3**	K18-	99%	$C_{125}H_{232}N_{50}O_{36}$	3009.761	3009.786	20%
**S4**	K18ac	>90%	$C_{127}H_{234}N_{50}O_{37}$	3051.800	3051.797	6%
**S5**	K18dec	90%	$C_{135}H_{250}N_{50}O_{37}$	3163.920	3163.922	6%
**S6**	K18MTU	97%	$C_{127}H_{235}N_{51}O_{36}S$	3082.758	3082.785	3%
**S7**	K18DTU	99%	$C_{136}H_{253}N_{51}O_{36}S$	3208.900	3208.926	4%
**S8**	K36ac	94%	$C_{87}H_{142}N_{30}O_{22}S$	1991.065	1991.064	30%
**S9**	K36dec	84%	$C_{95}H_{158}N_{30}O_{22}S$	2103.194	2103.189	22%
**S10**	K36MTU	83%	$C_{87}H_{143}N_{31}O_{21}S_2$	2021.722	2022.052	16%
**S11**	K36DTU	92%	$C_{96}H_{161}N_{31}O_{21}S_2$	2147.841	2148.192	23%

*Peptide purity measured by integration of HPLC chromatograms at 214 nm. See Supplementary Fig. 20 for HPLC traces.

Semi-synthesis of modified full-length histone H3

Histone H3 semi-synthesis was modified from the protocol by Guidotti *et al.*, 2020[4].

Peptide hydrazides (3.0 equiv.) were converted into peptide thioesters by *in situ* pyrazole formation, as reported[7]. Lyophilized peptides were dissolved in a dispersion of 4-mercaptophenylacetic acid (MPAA, 60 equiv.) in acidic ligation buffer (6 M guanidinium

chloride, 0.2 M NaH_2PO_4, 0.2 M MPAA, pH 3) to a concentration of 10 mM, followed by addition of acetylacetone (0.5 M in H_2O, 7.5 equiv.) and stirring at room temperature for 2-4 h under Ar atmosphere. The reaction was followed by HPLC-MS and, when full conversion to the MPAA thioester was detected, the crude mixtures were stored at -20 ºC for ligation within the following days (Supplementary Fig. 16).

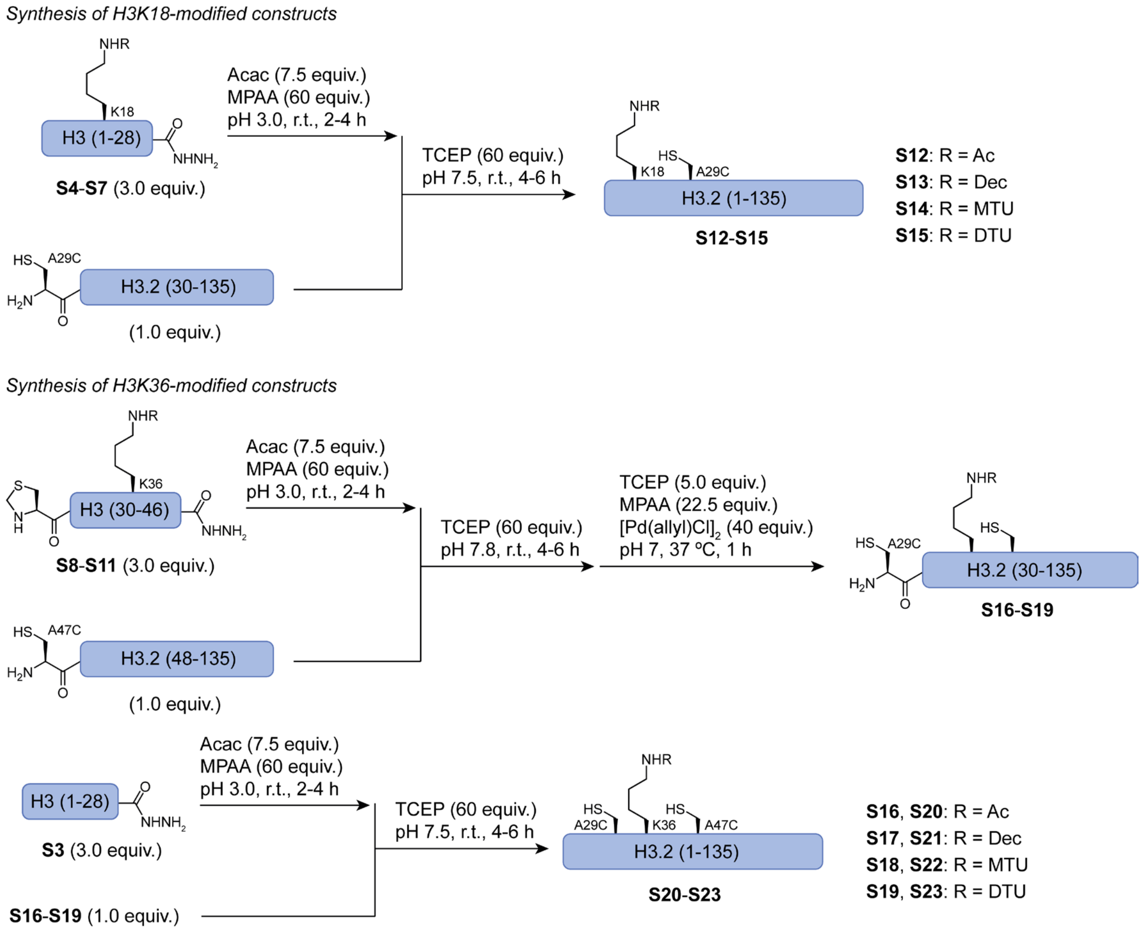

Supplementary Fig. 16. Summary of histone semi-synthesis methods. Acac: acetylacetone.

Peptide thioesters were linked to N-terminal Cys-containing histone fragments (see recombinant expression and purification below) by native chemical ligation. The histone fragment lyophilized powder (1.0 equiv.) was dissolved in the peptide thioester crude mixture (3.0 equiv.) by vortexing, and the concentration of histone fragment was adjusted to ~1 mM with ligation buffer (6 M guanidinium chloride, 0.2 M NaH_2PO_4, pH 7). Then, tris(2-carboxyethyl)phosphine hydrochloride (TCEP, 0.5 M in ligation buffer, 60 equiv.) was added, the pH was adjusted carefully to 7.8 (for ligation to H3(47-135) fragments) or 7.5 (for ligation to H3(29-135) fragments) using NaOH (5 M or 1 M in H_2O), and the mixture was allowed to stir at room temperature for 4-6 h under Ar atmosphere. Reactions were followed by HPLC-MS and analytical HPLC and, either diluted with eluents III and IV and purified directly by semi-preparative HPLC for full-length constructs, or treated for thiazolidine deprotection in the case of ligation to H3(47-135) fragments (Supplementary Fig. 16).

Thiazolidine deprotection was achieved by addition of TCEP (0.5 M in ligation buffer, 5.0 equiv.), MPAA (0.2 M in ligation buffer, 22.5 equiv.) and $[Pd(allyl)Cl]_2$ (0.1 M in ligation buffer, 40 equiv.) to the crude ligation mixture, and vigorous shaking at 37 °C for 1 h under Ar atmosphere (Supplementary Fig. 16). Thereafter, DTT (0.5 M in H_2O) was added to final concentration of 250 mM, the mixture was vortexed extensively, and it was further diluted to half with H_2O/MeCN 1:1 before centrifugation (21130 g, 10 min, 25 °C). The pellet was washed twice with DTT (250 mM in H_2O/MeCN 3:1 with 0.05% TFA), and the combined supernatant was diluted with eluents III and IV and purified by semi-preparative HPLC.

The following full-length histones were obtained (Supplementary Table 7):

Supplementary Table 7. Histone analyses and synthetic yields.

Protein	Modification	Purity*	Formula	HRMS*	Calcd. MW	Synth. yield
**S12**	K18ac	>85%	$C_{671}H_{1131}N_{215}O_{187}S_3$	15299.6	15299.0	34%
**S13**	K18dec	>90%	$C_{679}H_{1147}N_{215}O_{187}S_3$	15411.2	15411.1	36%
**S14**	K18MTU	>95%	$C_{671}H_{1132}N_{216}O_{186}S_4$	15330.0	15330.0	42%
**S15**	K18DTU	>95%	$C_{680}H_{1150}N_{216}O_{186}S_4$	15456.2	15456.2	42%
**S20**	K36ac	97%	$C_{671}H_{1131}N_{215}O_{187}S_4$	15330.8	15331.0	12%
**S21**	K36dec	92%	$C_{679}H_{1147}N_{215}O_{187}S_4$	15443.0	15443.2	19%
**S22**	K36MTU	>80%	$C_{671}H_{1132}N_{216}O_{186}S_5$	15362.0	15362.1	22%
**S23**	K36DTU	>85%	$C_{680}H_{1150}N_{216}O_{186}S_5$	15488.0	15488.3	13%

*Protein purity measured by integration of HPLC chromatograms at 214 nm. HRMS calculated by molecular weight deconvolution with MassLynx MaxEnt1. See Supplementary Figs. 21, 24, 25 for HPLC traces and mass spectra.

Histone expression and purification

Expression and purification of H3(29-135) and H3(47-135) constructs

H3.2 C110A fragment expression and purification were performed based on the protocol by Guidotti *et al.*, 2020[4].

E. coli BL21 (DE3) cells were transformed with a pET30 plasmid encoding for the corresponding truncated histone H3.2 with an N-terminal A→C mutation, an N-terminal fusion of 6xH-SUMO, and the mutation C110A common in H3 protein semi-synthesis. Cells were cultured in 5 L LB medium with 50 µg/mL kanamycin at 37 °C with shaking at 200 rpm until OD_{600} = 0.6-0.8. Then, 1 M isopropyl β-D-1-thiogalactopyranoside (IPTG) was added to a concentration of 150 mM and the cells were cultured for additional 4 h at 37 °C. Cell pellets were obtained by centrifugation (4000 g, 20 min, 4 °C), suspended in H3 lysis buffer (60 mL, 20 mM Tris-HCl, 200 mM NaCl, 1 mM EDTA, 1 mM 2-mercaptoethanol, pH 7.5) supplemented with protease inhibitors (cOmplete EDTA free, Merck), and flash-frozen for storage at -70 °C. Thereafter, the suspension was thawed and lysed by sonication for 10 min (15 s on, 45 s off pulses). The insoluble fraction was collected by centrifugation (15000 g, 30 min, 4 °C), washed with standard H3 lysis buffer (2 × 60 mL), with H3 lysis buffer supplemented with 1% Triton X-100 (1 × 60 mL), and with standard H3 lysis buffer (1 × 60 mL). The resulting pellet was suspended in a solubilization buffer (6 M guanidinium chloride, 50 mM Tris-HCl, 100 mM NaCl, 5 mM imidazole, 1 mM 2-mercaptoethanol, pH 7.5) by

vigorous shaking and stirring for 2-4 h at 4 ºC, centrifuged (15000 g, 30 min, 4 ºC), and the supernatant was purified in several batches by Ni-NTA FPLC. The column was washed with buffer E (6 M urea, 150 mM Tris-HCl, 150 mM NaCl, 50 mM imidazole, pH 7.5), and the sample was eluted by a mixture 60:40 (v/v) of buffer E and buffer F (6 M urea, 150 mM Tris-HCl, 150 mM NaCl, 500 mM imidazole, pH 7.5). Fractions containing the corresponding 6x-SUMO-H3 construct were identified by SDS-PAGE (15% acrylamide gel), combined, mixed with commercial Ulp1 SUMO protease (Merck, cat. # SAE0067) and dialyzed against 2 L of a cleavage buffer (1 M urea, 75 mM Tris-HCl, 150 mM NaCl, 5 mM 1,4-dithiothreitol (DTT), 25 mM L-arginine, pH 7.5). SUMO cleavage was verified by HPLC-MS, and the sample was dialyzed into 1% AcOH and lyophilized. The resulting powder was either treated with $[Pd(allyl)Cl]_2$, MPAA and TCEP (in the case of thiazolidine formation due to formaldehyde contamination) and purified, or purified directly by preparative HPLC. Typical yields were of 2.5-5 mg of lyophilized protein powder per liter of bacterial culture.

All construct sequences can be found in Supplementary Table 5.

Expression and purification of wild type H2A, H2B, H3 and H4

Wild type H2A, H2B, and H4, and H3 C110A, were produced based on literature procedures[8].

Transformed *E.coli* BL21 (DE3) cells were cultured in 6 L LB medium with 100 µg/mL ampicillin at 37 ºC with shaking at 200 rpm until OD_{600} of ~0.6-0.8. Then, 1 M IPTG was added to a final concentration of 1 mM, and the cells were cultured for additional 3-4 h at 37 ºC. Cells were harvested, lysed, and the pellets were prepared as mentioned before for 6xH-SUMO-H3 constructs. Pellets were then resuspended in a solubilization buffer (6 M guanidinium chloride, 20 mM Tris-HCl, 1 mM EDTA, 1 mM 2-mercaptoethanol, pH 7.5) by stirring at 4 ºC overnight, centrifuged (15000 g, 30 min, 4 ºC), and the supernatant was dialyzed against 2 L of a urea-containing buffer (7 M urea, 10 mM Tris-HCl, 100 mM NaCl, 1 mM EDTA, 5 mM 2-mercaptoethanol, 0.2 mM PMSF, pH 7.5) and centrifuged again (15000 g, 30 min, 4 ºC). The new supernatant was purified by IEX FPLC using a 0→100% linear gradient of NaCl between the previous urea-containing buffer (100 mM NaCl) and high-salt version of the same buffer (1500 mM NaCl). Fractions containing the desired histone were identified by SDS-PAGE (15% acrylamide gel), combined, and purified by preparative HPLC. Typical yields were of 7-25 mg of lyophilized protein powder per liter of bacterial culture.

All sequences can be found in Supplementary Table 5.

Gel electrophoresis of protein, DNA and nucleosome samples

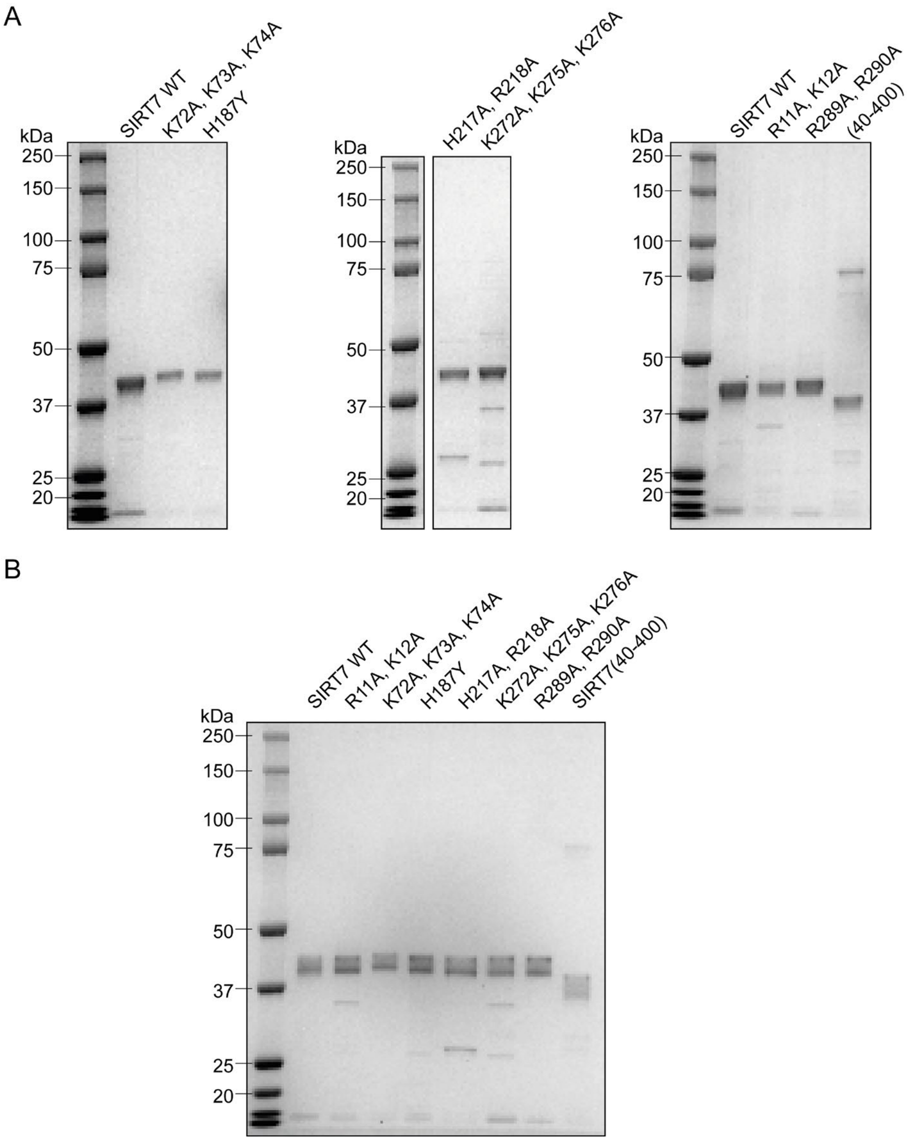

Supplementary Fig. 17. SDS-PAGE analysis of recombinant SIRT7 preparations employed in this work. **A**, Example of SDS-PAGE analysis of each of the constructs stained with Coomasie Blue, as used for SIRT7 quantification. **B**, Summary gel containing ~0.3 µg of each SIRT7 construct, stained with Coomasie Blue. See Supplementary Figs. 22–23 for mass spectra.

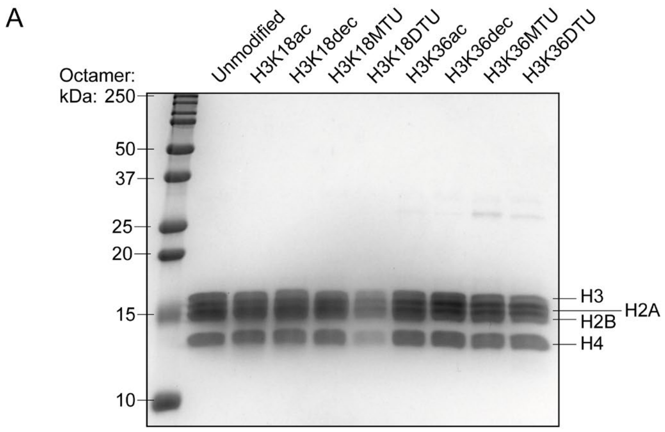

Supplementary Fig. 18. Materials for nucleosome preparation. A, SDS-PAGE analysis of all histone octamers employed in this work. Octamers are labelled according to H3 modifications, as they all contain wild type H2A, H2B and H4. Unmodified octamers contain H3.2 C110A. **B**, Agarose gel electrophoretic analysis of DNA samples, stained with GelRed.

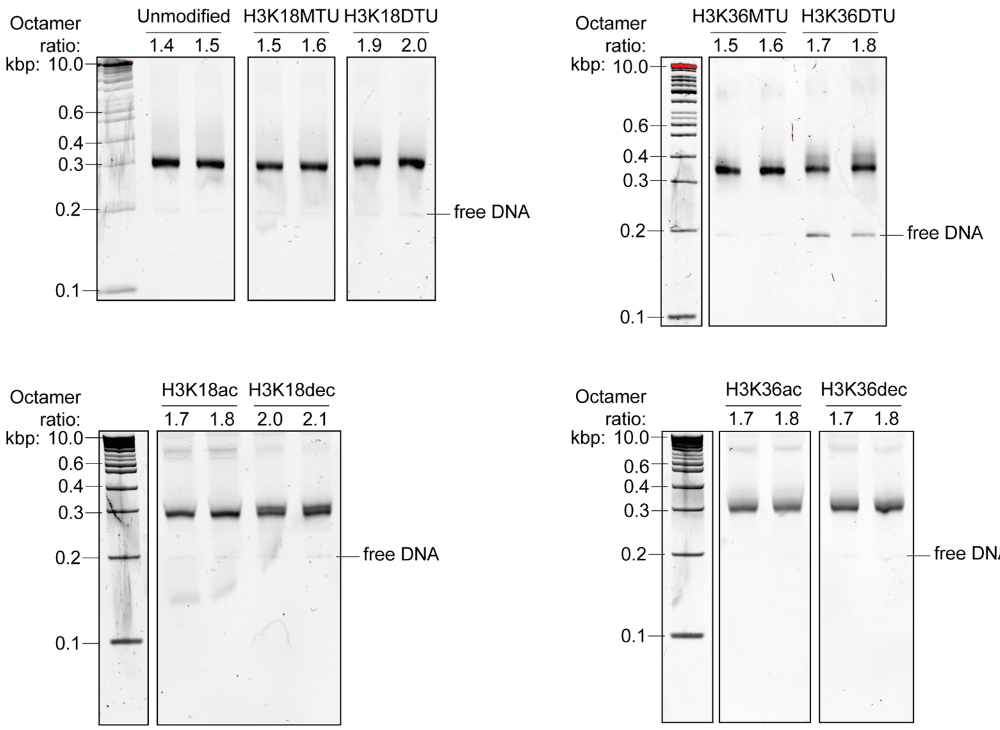

Supplementary Fig. 19. Examples of native gel electrophoretic analysis of nucleosome samples, stained with GelRed.

Purity traces

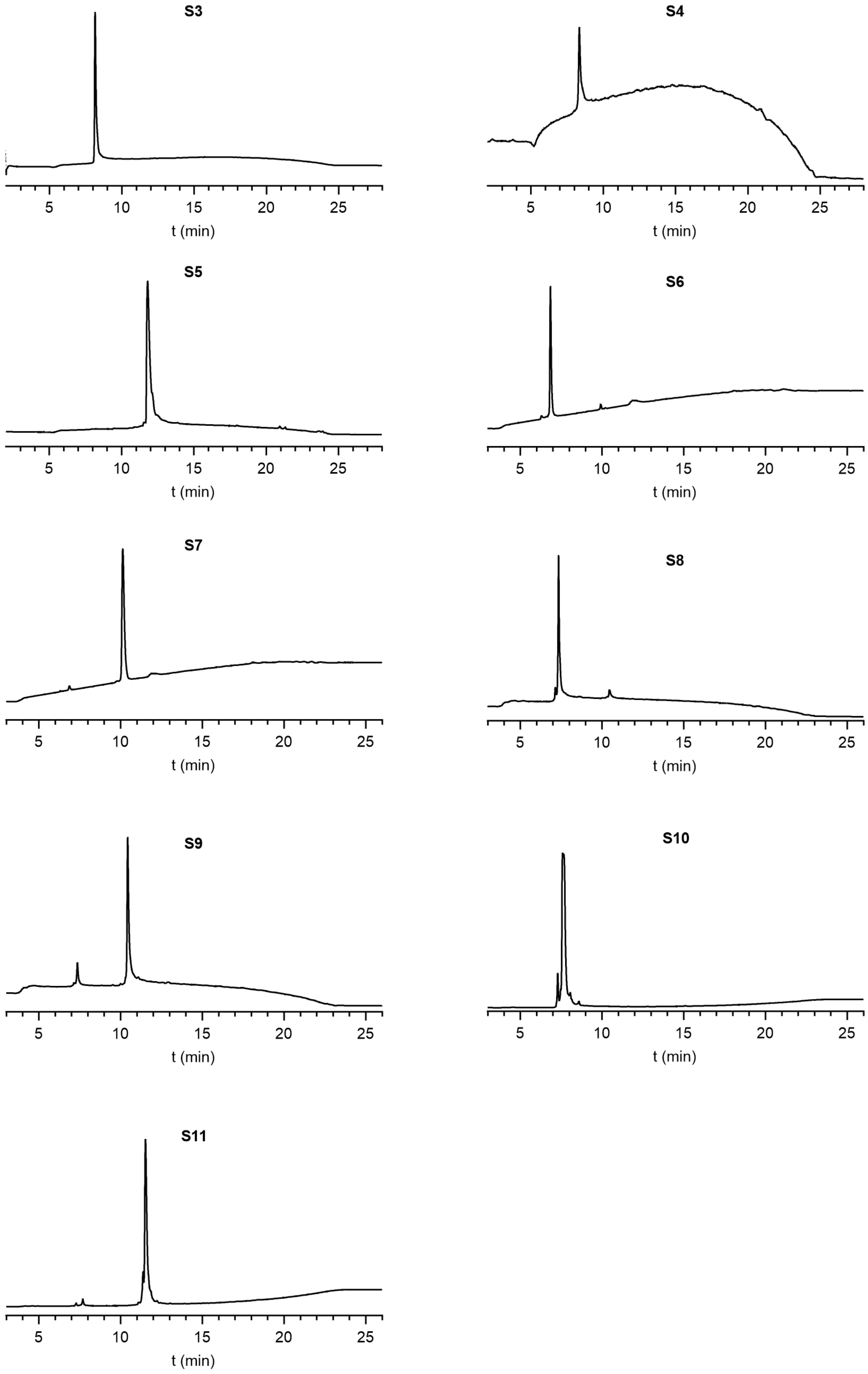

Supplementary Fig. 20. Peptide HPLC traces.

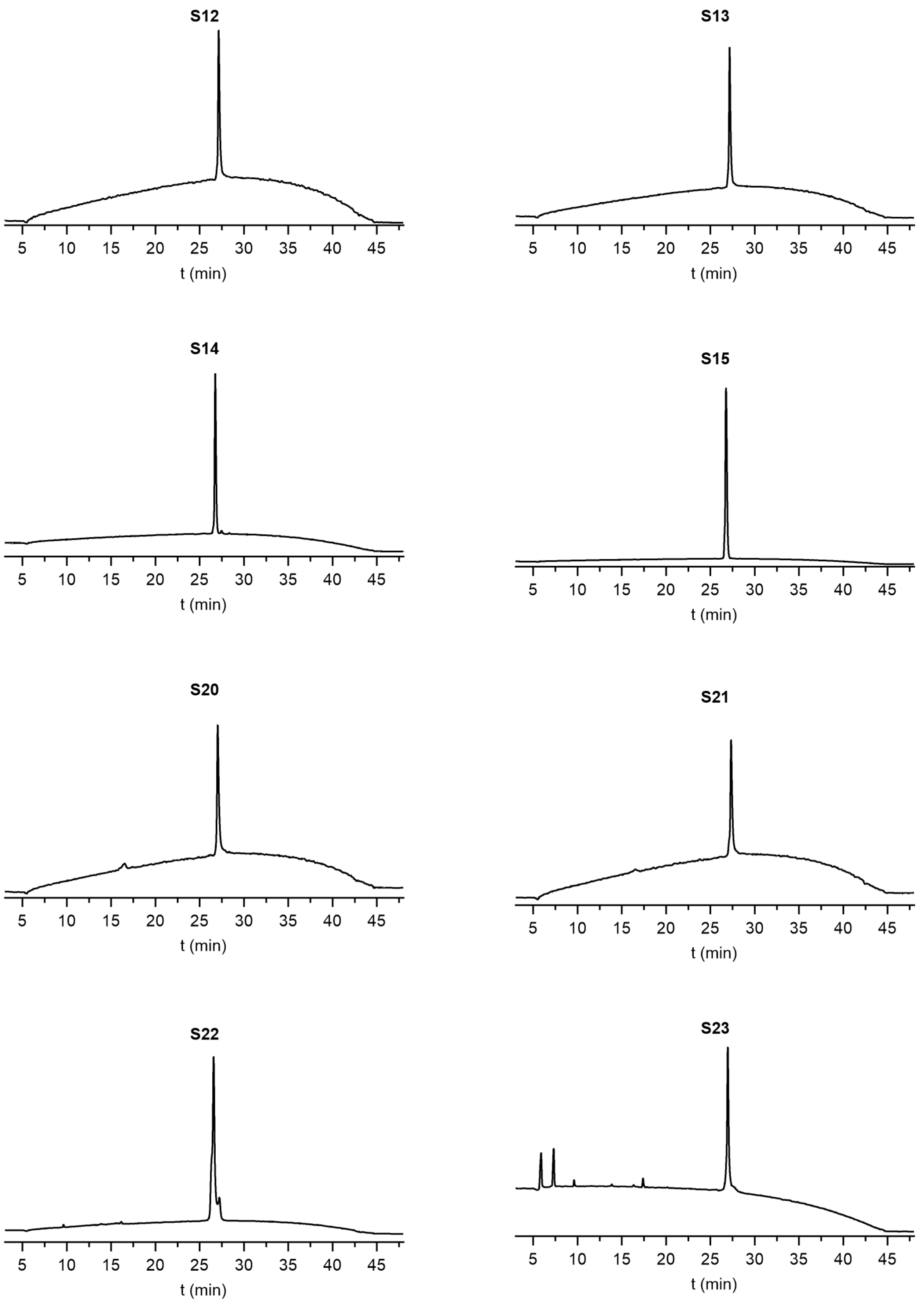

Supplementary Fig. 21. Semi-synthetic H3 HPLC traces. Note: compound **S23** was injected dissolved in unfolding buffer, which gave extra signals between 5 and 8 min.

Mass spectra

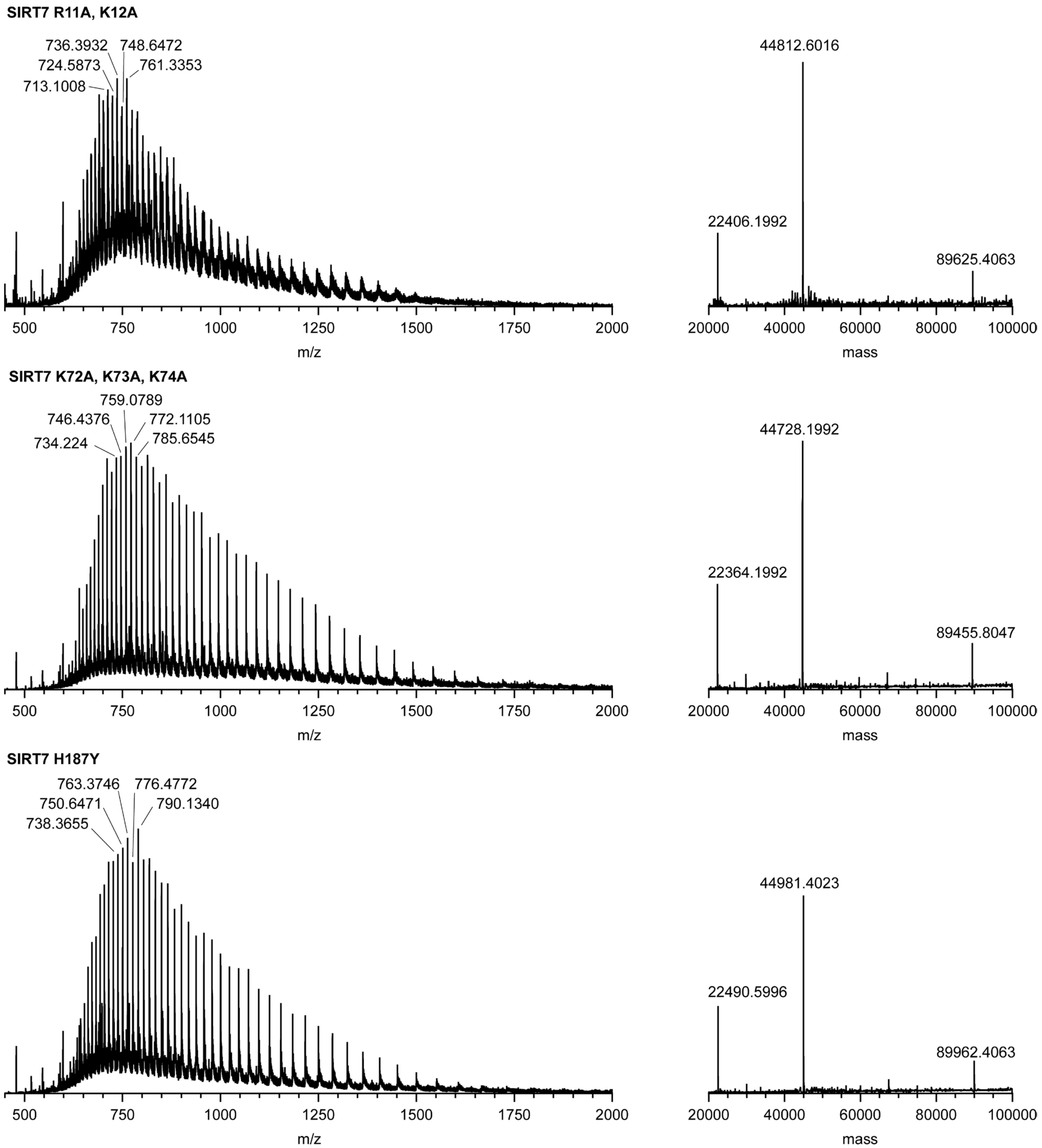

Supplementary Fig. 22. SIRT7 mass spectra (left) and deconvoluted mass (right).

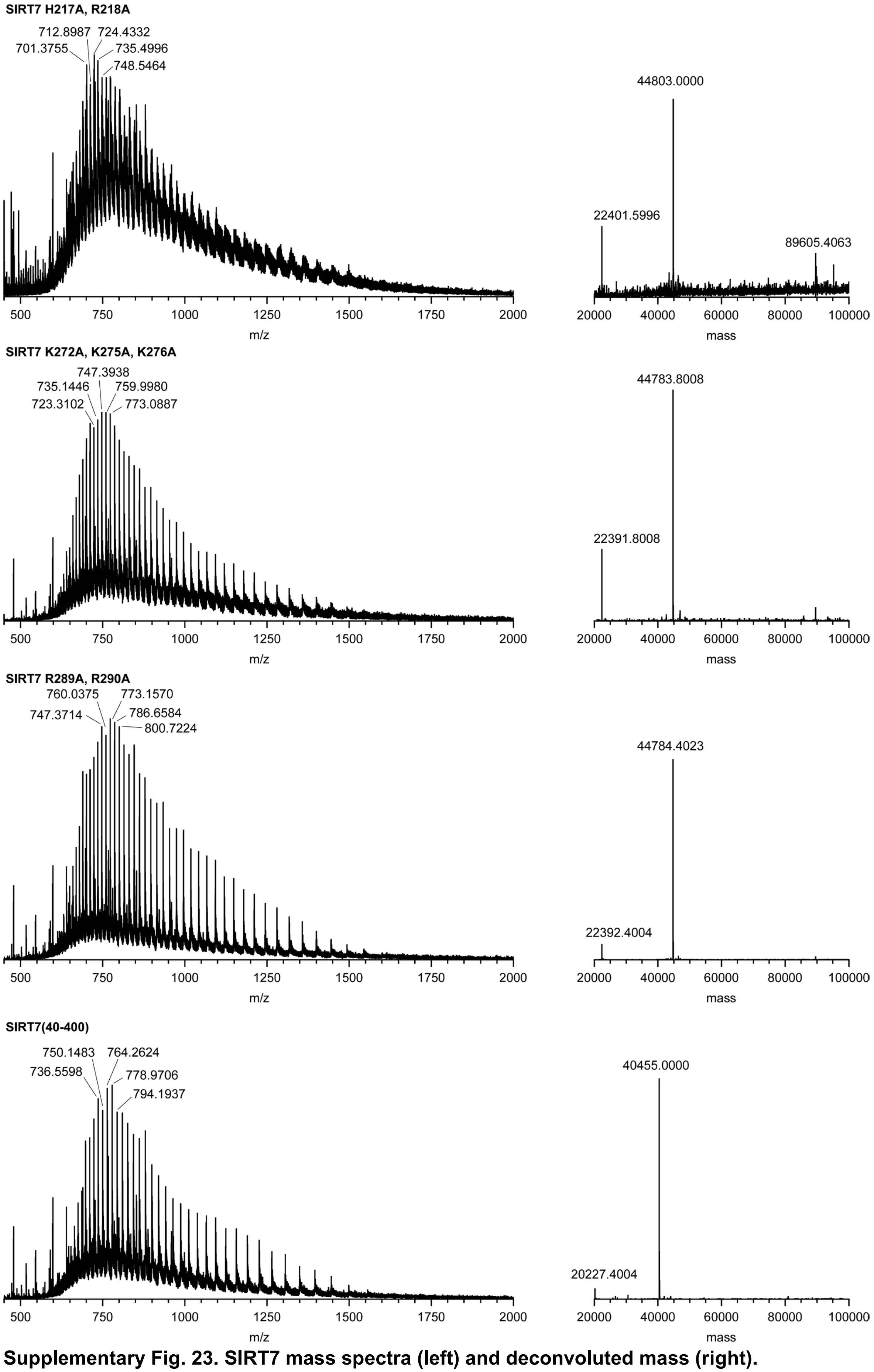

Supplementary Fig. 23. SIRT7 mass spectra (left) and deconvoluted mass (right).

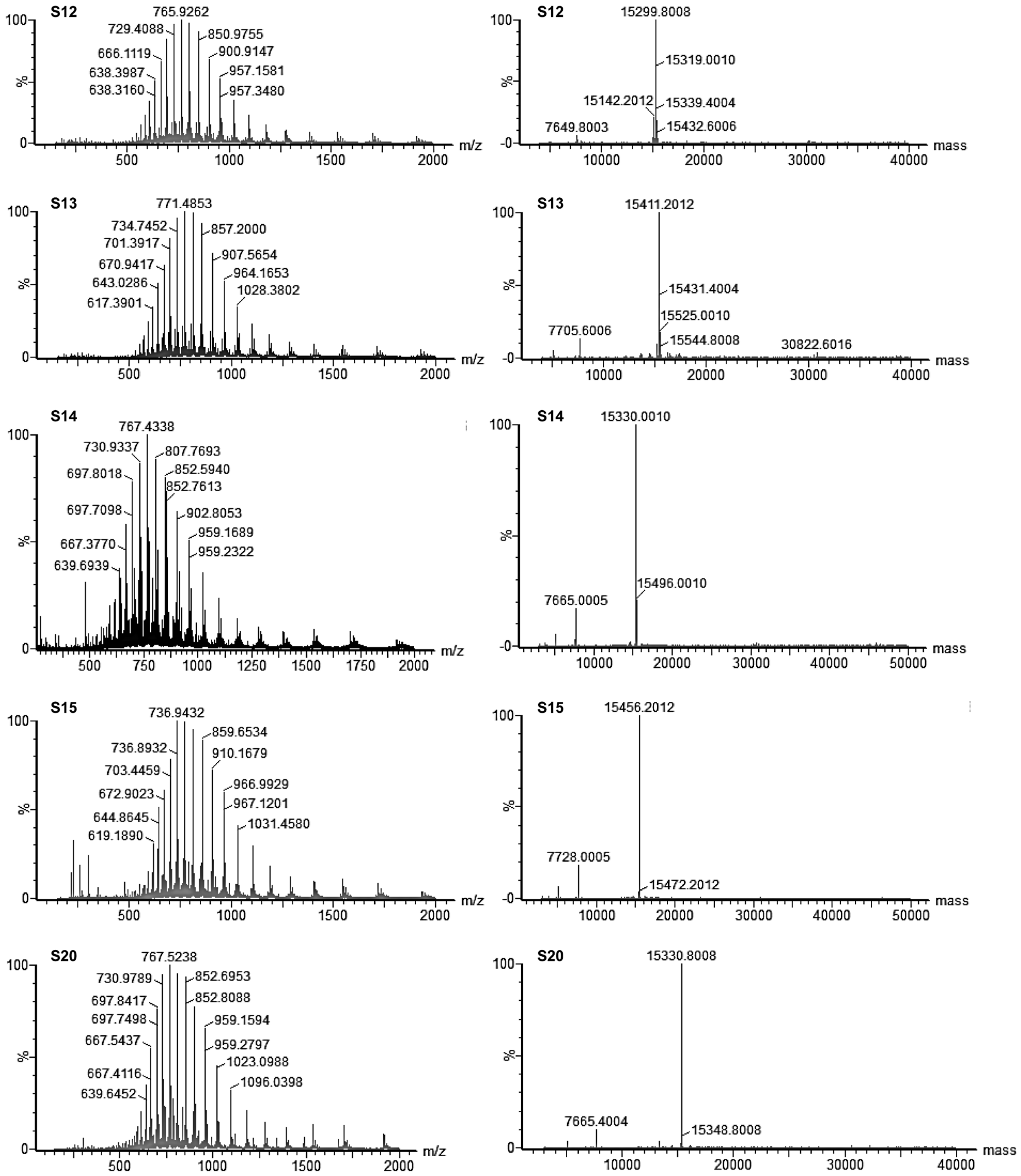

Supplementary Fig. 24. Full-length modified histone H3 mass spectra (left) and deconvoluted mass (right).

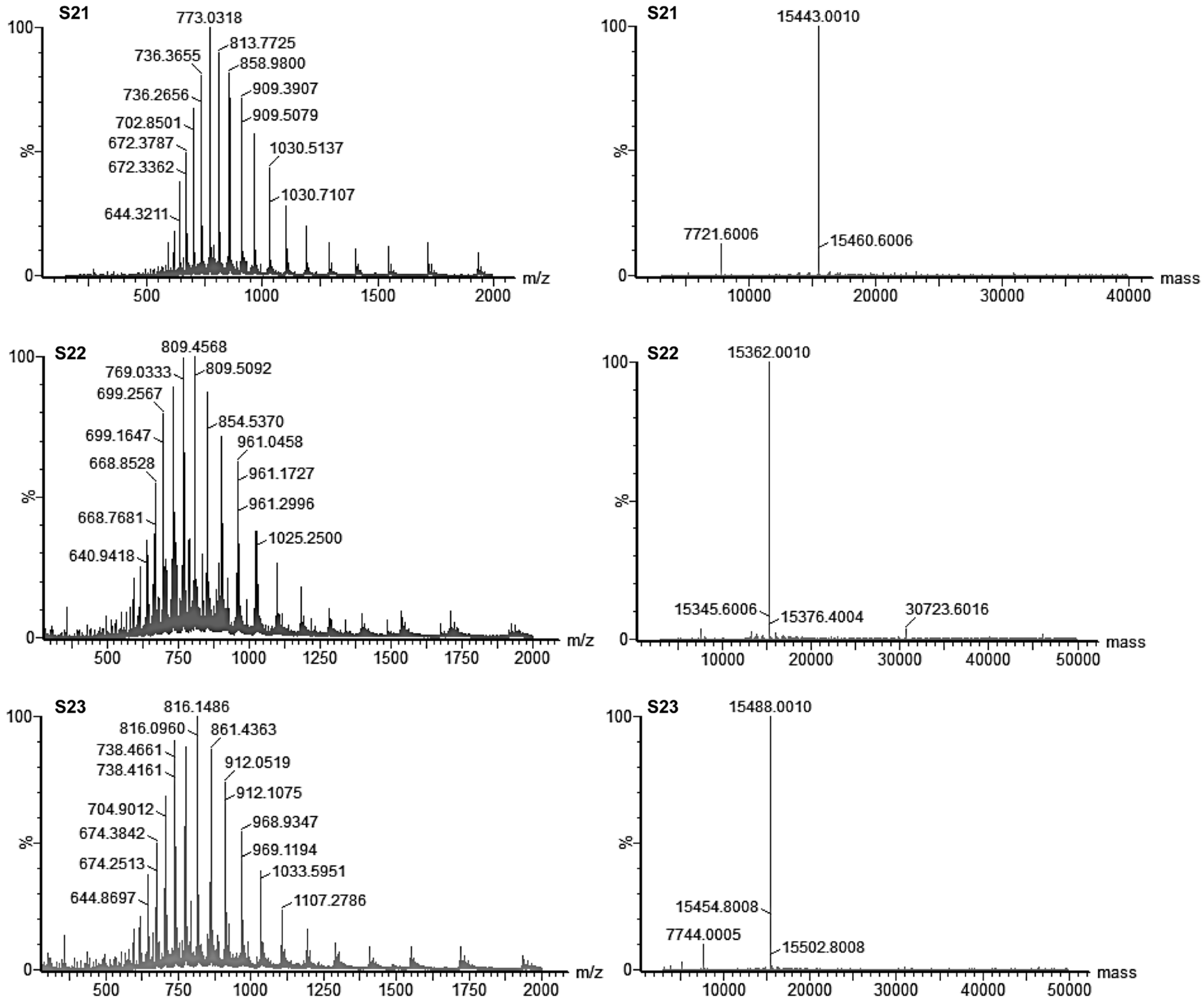

Supplementary Fig. 25. Full-length modified histone H3 mass spectra (left) and deconvoluted mass (right).

Supplementary References

1. Wang, H., Farnung, L., Dienemann, C. & Cramer, P. Structure of H3K36-methylated nucleosome-PWWP complex reveals multivalent cross-gyre binding. *Nat Struct Mol Biol* **27**, 8-13 (2020).
2. Elfmann, C. & Stulke, J. PAE viewer: a webserver for the interactive visualization of the predicted aligned error for multimer structure predictions and crosslinks. *Nucleic Acids Res* **51**, W404-W410 (2023).
3. Tong, Z. et al. SIRT7 Is an RNA-Activated Protein Lysine Deacylase. *ACS Chem Biol* **12**, 300-310 (2017).
4. Guidotti, N. & Fierz, B. Semisynthesis and Reconstitution of Nucleosomes Carrying Asymmetric Histone Modifications. *Methods Mol Biol* **2133**, 263-291 (2020).
5. Eissler, S. et al. Substitution determination of Fmoc-substituted resins at different wavelengths. *J Pept Sci* **23**, 757-762 (2017).
6. Troelsen, K.S. et al. Mitochondria-targeted inhibitors of the human SIRT3 lysine deacetylase. *RSC Chem Biol* **2**, 627-635 (2021).
7. Flood, D.T. et al. Leveraging the Knorr Pyrazole Synthesis for the Facile Generation of Thioester Surrogates for use in Native Chemical Ligation. *Angew Chem Int Ed Engl* **57**, 11634-11639 (2018).
8. Dyer, P.N. et al. Reconstitution of nucleosome core particles from recombinant histones and DNA. *Methods Enzymol* **375**, 23-44 (2004).
