# Supplementary material for: Structural basis of SIRT7 nucleosome engagement and substrate specificity
Source: Nat Commun. 2025 Feb 4;16:1328. doi: 10.1038/s41467-025-56529-y (PMC11790868; doi:10.1038/s41467-025-56529-y)
Supplement: Supplementary file 2 — Description of Additional Supplementary Files [file 41467_2025_56529_MOESM2_ESM.pdf]

Description of Additional Supplementary Files

Supplementary Data 1. Plasmid sequences. Sequence of plasmids for 6xH-SIRT7 expression in *E. coli* and for SIRT7-mCherry-HA expression in mammalian cells.
