# Supplementary material for: Structural basis of SIRT7 nucleosome engagement and substrate specificity
Source: Nat Commun. 2025 Feb 4;16:1328. doi: 10.1038/s41467-025-56529-y (PMC11790868; doi:10.1038/s41467-025-56529-y)
Supplement: Supplementary file 4 — Reporting Summary [file 41467_2025_56529_MOESM4_ESM.pdf]

nature portfolio

Corresponding author(s): Prof. Beat Fierz
Dr. Carlos Moreno-Yruela

Last updated by author(s): Jan 13, 2025

# Reporting Summary

Nature Portfolio wishes to improve the reproducibility of the work that we publish. This form provides structure for consistency and transparency in reporting. For further information on Nature Portfolio policies, see our Editorial Policies and the Editorial Policy Checklist.

## Statistics

For all statistical analyses, confirm that the following items are present in the figure legend, table legend, main text, or Methods section.

| n/a | Confirmed | |
|---|---|---|
| ☐ | ☒ | The exact sample size (*n*) for each experimental group/condition, given as a discrete number and unit of measurement |
| ☐ | ☒ | A statement on whether measurements were taken from distinct samples or whether the same sample was measured repeatedly |
| ☐ | ☒ | The statistical test(s) used AND whether they are one- or two-sided<br>*Only common tests should be described solely by name; describe more complex techniques in the Methods section.* |
| ☒ | ☐ | A description of all covariates tested |
| ☐ | ☒ | A description of any assumptions or corrections, such as tests of normality and adjustment for multiple comparisons |
| ☐ | ☒ | A full description of the statistical parameters including central tendency (e.g. means) or other basic estimates (e.g. regression coefficient) AND variation (e.g. standard deviation) or associated estimates of uncertainty (e.g. confidence intervals) |
| ☐ | ☒ | For null hypothesis testing, the test statistic (e.g. *F*, *t*, *r*) with confidence intervals, effect sizes, degrees of freedom and *P* value noted<br>*Give P values as exact values whenever suitable.* |
| ☒ | ☐ | For Bayesian analysis, information on the choice of priors and Markov chain Monte Carlo settings |
| ☒ | ☐ | For hierarchical and complex designs, identification of the appropriate level for tests and full reporting of outcomes |
| ☒ | ☐ | Estimates of effect sizes (e.g. Cohen's *d*, Pearson's *r*), indicating how they were calculated |

*Our web collection on statistics for biologists contains articles on many of the points above.*

## Software and code

Policy information about availability of computer code

| | |
|---|---|
| Data collection | Cryo-EM image processing was performed using cryoSPARC v4.2<br>Gel image processing was performed using ImageJ v1.53 and v1.54<br>Cell image processing was performed using ImageJ Macro in Fiji v2.14.0 |
| Data analysis | Structural alignments and superpositions were performed using UCSF ChimeraX v1.4 and PyMOL v1.8.2.0<br>Atomic models were built using Coot v0.9.4 and refined using Phenix v1.19.2-4158<br>Cell imaging data was processed with RStudio vR-4.4.1<br>Data groups were analysed and plotted using GraphPad Prism v10 |

For manuscripts utilizing custom algorithms or software that are central to the research but not yet described in published literature, software must be made available to editors and reviewers. We strongly encourage code deposition in a community repository (e.g. GitHub). See the Nature Portfolio guidelines for submitting code & software for further information.

## Data

Policy information about availability of data

All manuscripts must include a data availability statement. This statement should provide the following information, where applicable:

- Accession codes, unique identifiers, or web links for publicly available datasets
- A description of any restrictions on data availability
- For clinical datasets or third party data, please ensure that the statement adheres to our policy

The reconstructed cryoEM maps and atomic models generated in this study have been deposited in the EMDB and PDB databases, under accession codes EMD-51453 [https://www.ebi.ac.uk/pdbe/entry/emdb/EMD-51453] and PDB 9GMR [https://doi.org/10.2210/pdb9gmr/pdb] for SIRT7:H3K36MTU, and under accession codes EMD-51449 [https://www.ebi.ac.uk/pdbe/entry/emdb/EMD-51449] and PDB 9GMK [https://doi.org/10.2210/pdb9gmk/pdb] for SIRT7:H3K18DTU. The micrograph images data have been deposited in the EMPIAR database under accession code EMPIAR-12525. All other data generated and analysed in this study are provided in the Supplementary Information/Source Data file. Plasmid sequences are provided as separate files.

## Research involving human participants, their data, or biological material

Policy information about studies with human participants or human data. See also policy information about sex, gender (identity/presentation), and sexual orientation and race, ethnicity and racism.

| | |
|---|---|
| Reporting on sex and gender | N/A |
| Reporting on race, ethnicity, or other socially relevant groupings | N/A |
| Population characteristics | N/A |
| Recruitment | N/A |
| Ethics oversight | N/A |

Note that full information on the approval of the study protocol must also be provided in the manuscript.

# Field-specific reporting

Please select the one below that is the best fit for your research. If you are not sure, read the appropriate sections before making your selection.

☒ Life sciences ☐ Behavioural & social sciences ☐ Ecological, evolutionary & environmental sciences

For a reference copy of the document with all sections, see nature.com/documents/nr-reporting-summary-flat.pdf

# Life sciences study design

All studies must disclose on these points even when the disclosure is negative.

| | |
|---|---|
| Sample size | The experiments were performed in independent replicates and sample sizes as indicated. No sample size calculation was performed, and experiments were always performed at least in triplicate |
| Data exclusions | No data was excluded from analysis |
| Replication | All experiments were repeated multiple times on different days and using new reagent dilutions |
| Randomization | Samples were allocated randomly |
| Blinding | No blinding was performed, as samples were measured in bulk without a subjective component |

# Reporting for specific materials, systems and methods

We require information from authors about some types of materials, experimental systems and methods used in many studies. Here, indicate whether each material, system or method listed is relevant to your study. If you are not sure if a list item applies to your research, read the appropriate section before selecting a response.

## Materials & experimental systems

| n/a | Involved in the study |
|---|---|
| ☐ | ☒ Antibodies |
| ☐ | ☒ Eukaryotic cell lines |
| ☒ | ☐ Palaeontology and archaeology |
| ☒ | ☐ Animals and other organisms |
| ☒ | ☐ Clinical data |
| ☒ | ☐ Dual use research of concern |
| ☒ | ☐ Plants |

## Methods

| n/a | Involved in the study |
|---|---|
| ☒ | ☐ ChIP-seq |
| ☒ | ☐ Flow cytometry |
| ☒ | ☐ MRI-based neuroimaging |

# Antibodies

Antibodies used

H3K18ac Rabbit, ThermoFisher Scientific #703896
H3K36ac Rabbit, Sigma-Aldrich #SAB5600229
H3 Mouse, Cell Signaling Technology #3638
SIRT7 Rabbit, Cell Signaling Technology #5360
mCherry Rabbit, Proteintech #26765-1-AP
GAPDH Rabbit, Cell Signaling Technology #2118
H3K18ac Rabbit, ThermoFisher Scientific #MA5-24669
H3K36ac Rabbit, ThermoFisher Scientific #MA5-24672
H4K16ac Rabbit, ThermoFisher Scientific #MA5-34717

Validation

All primary antibodies were previously validated for Western blot or immunofluorescence, and here positive and negative controls were included on each experiment

# Eukaryotic cell lines

Policy information about cell lines and Sex and Gender in Research

Cell line source(s)

HEK293F cells with stable SIRT7 knockout were obtained from the laboratory of Alejandro Vaquero (Josep Carreras Leukemia Research Institute), which generated it and published it prior to our study (Simonet et al., 2020).

Authentication

No cell authentication was performed

Mycoplasma contamination

Tests were carried out by Microsynth (Mycoplasma Testing for Cell Culture service) regularly and after each round of experiments

Commonly misidentified lines
(See ICLAC register)

No commonly misidentified cell lines were used

# Plants

Seed stocks

*Report on the source of all seed stocks or other plant material used. If applicable, state the seed stock centre and catalogue number. If plant specimens were collected from the field, describe the collection location, date and sampling procedures.*

Novel plant genotypes

*Describe the methods by which all novel plant genotypes were produced. This includes those generated by transgenic approaches, gene editing, chemical/radiation-based mutagenesis and hybridization. For transgenic lines, describe the transformation method, the number of independent lines analyzed and the generation upon which experiments were performed. For gene-edited lines, describe the editor used, the endogenous sequence targeted for editing, the targeting guide RNA sequence (if applicable) and how the editor was applied.*

Authentication

*Describe any authentication procedures for each seed stock used or novel genotype generated. Describe any experiments used to assess the effect of a mutation and, where applicable, how potential secondary effects (e.g. second site T-DNA insertions, mosiacism, off-target gene editing) were examined.*
